# Black Chokeberry *Aronia Melanocarpa* L.—A Qualitative Composition, Phenolic Profile and Antioxidant Potential

**DOI:** 10.3390/molecules24203710

**Published:** 2019-10-15

**Authors:** Andrzej Sidor, Anna Gramza-Michałowska

**Affiliations:** Department of Gastronomy Sciences and Functional Foods, Faculty of Food Science and Nutrition, Poznań University of Life Sciences, Wojska Polskiego 31, 60624 Poznań, Poland; andrzej.sidor@up.poznan.pl

**Keywords:** chokeberry, *Aronia melanocarpa*, polyphenols, antioxidants, free radicals

## Abstract

Black chokeberry (*Aronia melnocarpa*) is a source of many bioactive compounds with a wide spectrum of health-promoting properties. Fresh, unprocessed chokeberry fruits are rarely consumed due to their astringent taste, but they are used in the food industry for the production of juices, nectars, syrups, jams, preserves, wines, tinctures, fruit desserts, jellies, fruit teas and dietary supplements. Polyphenols are biofactors that determine the high bioactivity of chokeberries, some of the richest sources of polyphenols, which include anthocyanins, proanthocyanidins, flavonols, flavanols, proanthocyanidins, and phenolic acids. Chokeberry fruit and products have great antioxidant and health-promoting potential as they reduce the occurrence of free radicals. This publication reviewed the scientific research regarding the phenolic compounds and the antioxidant potential of chokeberry fruits, products and isolated compounds. These findings may be crucial in future research concerning chokeberry based functional food products. Chokeberry fruits can be considered as promising component of designed food with enhanced antioxidant potential. However, like other plants and medicinal products of natural origin, black chokeberry requires extensive studies to determine its antioxidant potential, safety and mechanisms of action.

## 1. Introduction

Black chokeberry (*Aronia melanocarpa* L.) is a perennial shrub of the *Rosaceae* family. The chokeberry plant is native to eastern parts of North America, but it was introduced in Europe at the beginning of the twentieth century. The usable parts of chokeberry are mainly fruits [1]. There are many cultivated chokeberry varieties: ‘Viking’, ‘Nero’, ‘Galicjanka’, ‘Hugin’, ‘N’, ‘Rubin’, ‘Aron’ and others [2,3,4,5]. Fresh, unprocessed chokeberry fruits are rarely consumed due to their astringent taste, but they are used in the food industry for the production of juices, nectars, syrups, jams, preserves, wines, tinctures, fruit desserts, jellies, fruit teas and dietary supplements [6]. Chokeberry anthocyanins can be added to foods as natural dyes [7,8]. The following paper provides a synthetic and systematic review of the current literature on the subject of selected antioxidative and bioactive compounds found in black chokeberry. The study involved a comparative analysis of the concentration of selected compounds in black chokeberry *Aronia melanocarpa* L. fruits, products and isolated compounds. The study involved a comparison of phenolics composition, e.g., anthocyanins, proanthocyanidins, flavonols, flavanols, proanthocyanidins, and phenolic acids. Additionally, the antioxidant activity of chokeberry fruits and products by mean of various assays had been reviewed. The latest findings in the subject have not been studied so far. The review’s findings may be crucial in future research concerning chokeberry based functional food products, a promising component of designed food with enhanced antioxidant potential.

## 2. Botanical and Chemical Characteristics

Chokeberry is a shrub which may grow as high as three meters. Young bushes are compact, whereas mature ones resemble a sprawling tree. Leaves covering branches are oval. They are green in the spring and summer, but in the autumn they become reddish brown. Fruits in clusters reach maturity in late August and September. Mature chokeberry fruits are black and blue from the outside and on the cross section. Fruits are spherical. Depending on the variety, their diameter ranges from 6.1 to 17.8 mm, whereas the weight of 100 fruits ranges from 32 to 111.7 g, sometimes even to about 280 g [1].

The composition and health value of chokeberry fruit depends on many factors, e.g., variety, maturity, environmental and climatic conditions [9,10]. The dry weight of chokeberry fruit, juice and pomace analysed by Mayer-Miebach, Adamiuk, and Behsnilian [11] was respectively 17.9–26%, 11.1–17.4% and 44.6–50%. Ochmian et al. [1,12] found that chokeberry fruits contained 15.3–19.5% of dry matter, including 14.2–18.7% of soluble substances. Skupień and Oszmiański [13] found high content of dry matter in fruit, i.e., 26.67–30.76%. The amount of protein in the fruit was low and amounted to 3.7 g/100 g DM (dry matter) of fruit [14]. Arginine, tyrosine, histidine, lysine, cysteine, α-alanine, asparagine, serine, glutamic acid and threonine are some of the amino acids in chokeberry. Most amino acids, including essential ones, were found in pomace. Their total weight amounted to 28.9 g/kg DM [15,16]. The total lipid content in fresh chokeberry fruit was 0.09–0.17% [17,18]. The largest amounts were found in stones and skin fractions, i.e., 13.9% and 2.9–9.8%, respectively [19]. The content of lipids in chokeberry pomace amounted to 5.5 g/100 g, including 65.0% of polyunsaturated fatty acids (PUFA). The pomace chiefly contained linoleic and oleic acids. Chokeberry seed oil contains sterols and phospholipids [20,21]. Sugars and dietary fibre are chokeberry carbohydrates. Studies have shown that the total content of carbohydrates in fresh fruits ranges from 6.21 to 20.92 g/100, total reducing sugars from 5.71 to 19.36 g/100 g, including fructose 1.38–4.71 g/100 g and glucose 1.09–5.70 g/100 g [1,11,13,22,23,24]. The content of sucrose ranges from 0 to 1.53 g/100 g of fruit. Chokeberry products and fruit contain relatively large amounts of sorbitol, i.e., 8.56 g/100 g of fruit. Chokeberry pomace, which consists of peels, stones and internal fruit cell structures, is a rich source of dietary fibre, which amounts to about 70% of dry matter.

More than 60% of chokeberry dietary fibre is composed of insoluble fraction (lignin, cellulose, hemicellulose) [25,26,27]. Chokeberries also contain vitamin B, carotenoids, tocopherols, vitamin C and vitamin K [17,21,28]. Ash amounts to 6.8 g/100 g DM in chokeberry fruit. Chokeberries also contain macroelements (K, Ca, P, Mg and Na), essential trace elements (Zn, Fe, Se, Cu, Mo, Cr), trace elements that are probably essential (Mn, Si, Ni, B, V) and toxic elements (Pb, Cd, Hg, As). Chokeberry fruit and products are potentially rich sources of K, Ca, P, Mg, Na, Fe and Zn [14,25,29]. Table 1 shows detailed data on the composition of chokeberry fruit and products.

## 3. Polyphenol Components

Polyphenols are biofactors that determine the high bioactivity of black chokeberries. Chokeberry fruits are some of the richest sources of polyphenols, which include anthocyanins, flavonols, flavanols, proanthocyanidins, and phenolic acids [33,50,51,52]. Various authors found the following content of polyphenols in chokeberry fruit: 7849 mg/100 g DM [53]; 6351,38 mg/100 g DM [54]; 37,600 mg/kg DM [55]; 1079–2996 mg Gallic Acid Equivalents/100 g fresh mass [56]; 819–1330 mg GAE/100 g FW (fresh weight) [1]; 778–1285 g GAE/kg FW [3]. Chokeberry products and waste are also rich in polyphenols [5,7,11,34,57,58]. The dark blue colour of chokeberry fruit is caused by the high concentration of anthocyanins, which include cyanidine 3-glucoside, 3-galactoside, 3-xyloside and 3-arabinoside. A small proportion of anthocyanins is attributed to pelargonidine-3-galactoside and pelargonidine-3-arabinoside [46,59,60]. Chokeberry flavonols are a diverse group of compounds, which mainly consist of quercetin derivatives. The main quercetin derivatives in chokeberries are: quercetin-3-glucoside, 3-galactoside, 3-rutinoside, 3-robinobioside and 3-vicianoside. Chokeberry fruit also contain flavonols in the form of isorhamnetin 3-galactoside, 3-glucoside, 3-neohesperidoside and 3-rutinoside; myricetin and kaempherol 3-galactoside and 3-glucoside [61,62,63]. The degree of proanthocyanidin polymerisation is a characteristic feature of chokeberries. Proanthocyanidins are composed of (−)-epicatechin and trace amounts of (+)-catechin, which may occur only at the end of a molecular chain of proanthocyanidins. Individual subunits are linked by C4 → C6 and C4 → C8 bonds [64,65,66].

The degree of proanthocyanidin polymerisation is DP > 10, which translates to the mean procyanidin polymerisation degree (mDP). The mDP of proanthocyanidin in fruits was 19–59; in juice it was 12–52 and in pomace it was 18–34 [11,13,40,45,53,65]. Hellström et al. [66] observed an exceptionally high degree of proanthocyanidin polymerisation in chokeberry juice and extract (mDP > 100). In chemical terms, proanthocyanidins are oligomers and polymers of flavan-3-ol, linked by B-type and A-type bonds. Therefore, the results of many studies have confirmed that the biological and chemical properties of proanthocyanidins depend on their structure, and in particular the molecular weight also expressed as the degree of polymerisation (DP). Part of (−)-epicatechin in chokeberry fruit has the form of monomers [53,59,67], while powders and chokeberry juice epicatechin can be found in combination with cyanidin glycosides [33]. 

Chokeberries mostly contain chlorogenic and neochlorogenic acids. Other phenolic acids are cryptochlorogenic acid, p-coumaric acid and its derivatives, caffeic acid and its derivatives, protocatechuic, vanillic, ferulic, salicylic, syringic, 4-hydroxybenzoic and ellagic acids. Dried juice also contains methyl esters of chlorogenic and neochlorogenic acids, 2,4,6-trihydroxybenzaldehyde, 3-hydroxybenzoic acid and phenylacetic acid derivatives [68]. Gentizinic and synapic acids were identified in chokeberry honey [69]. There were also reports on the content of eriodictyol 7-glucuronide; 3,7-diglycuronide, 7-xylose and naringenin-7-*O*-glucopyranoside and 7-*O*-β-d-glucopyranoside-5,7,3′,5′-tetrahydroxyflavanone, as flavanones found in chokeberry fruit and products [13,39,68,70,71,72]. Table 2 shows the polyphenol composition of chokeberry fruit and products.

## 4. Antioxidative Activity

Chokeberry fruits have high antioxidative potential, usually higher than other plant materials tested. The antioxidative activity of chokeberries was confirmed in various radical scavenging assays, the effects of transition metals on changes in the state of oxidation, and the ability to inhibit lipid peroxidation in a variety of model systems (Table 3). Tarko et al. [110] studied chokeberries, apples, plums, pears, bananas and melons. They found that chokeberry fruit components were the most active scavengers of the ABTS radical cation. The analysis of blackberry, blackcurrant, chokeberry, raspberry and redcurrant antioxidants (DPPH^•^) indicated a relatively high potential of chokeberries and selected blackcurrant varieties [120]. Nakajima, Tanaka, Seo, Yamazaki, and Saito [121] confirmed the DPPH radical scavenging ability using ethanol extracts of five berries, which were rich in anthocyanins: blackberries, black chokeberries, blackcurrants, bilberries and elderberries. The antioxidative activity of the chokeberry extract at the highest concentration (2 mg/mL) was lower than that of the other extracts, except the elderberry extract. Other studies tested the potential of 26 fruits to scavenge superoxide radicals (ROO^•^) in the ORAC assay. Chokeberry fruits were less active than elderberries and wild roses—their values amounted to 160.8, 205.4 and 201.1 µmol TE/g FW, respectively. The antiradical activity of the other fruits ranged from 2.3 to 98.9 µmol TE/g FW [36]. Wu et al. [59] evaluated the antioxidative potential of different fruits using ORAC for hydrophilic (H-ORAC) and lipophilic compounds (L-ORAC). Among 15 fruit samples, chokeberries and blackberries exhibited the highest antiradical activity. The H-ORAC values noted for chokeberries were many times greater than the L-ORAC values, i.e., 158.2 and 2.42 µmol TE/g FW, respectively.

The high antioxidative potential of chokeberry fruit was confirmed using ORAC, TRAP, hydroxyl radical (HO^•^) and nitric oxide (NO) assay. The ability of chokeberry extract to scavenge radicals (ROO^•^) in the ORAC assay amounted to 5165 µmol TE/g of the extract and it was only 10.7% lower than that of the elderberry extract. The TRAP assay showed that the ROO^•^ radical was more effectively scavenged by the chokeberry extract (4051 μmol TE/g extract) than the elderberry fruit extract (3230 μmol TE/g extract). The antiradical activity of the chokeberry, black elderberry and bilberry extracts against the hydroxyl radical HO^•^ did not differ significantly (1264–1293 μmol GAE/g extract). The chokeberry extract exhibited high nitrogen oxide (NO) scavenging capacity and inhibited the oxidation of α-linolenic acid [36]. The chokeberry fruits also inhibited liposomal oxidation effectively, exhibiting superior antioxidative properties to blackcurrants, rosehips and hawthorns [113].

Najda and Łabuda [73] investigated the antioxidative activity of different fruits and found that chokeberry fruits exhibited higher antioxidative activity than the other eight fruits. The activity was measured by determining the ability to reduce Fe in the FRAP assay and to scavenge the DPPH radical. The ABTS cation was effectively scavenged only by the ingredients of ‘Titania’ blackcurrants. The chokeberry fruit extracts exhibited antioxidative activity in DPPH, hydroxyl (HO•), superoxide anion (O_2_•^−^), and nitric oxide (NO) assay, and inhibited lipid oxidation. The ‘Viking’ and ‘Nero’ varieties had the greatest antioxidative potential, whereas the ‘Fertödi’ and ‘Aron’ cultivars exhibited the lowest potential [3]. Viskelis et al. [129] compared the antioxidative activity of different fruit varieties of chokeberries, raspberries and elderberries. The chokeberry extracts showed the highest antioxidative potential, where the ‘Viking’, ‘Aron’ and ‘Cleata’ varieties scavenged the DPPH radical comparably.

Apart from chokeberry fruit, fruit products and post-production waste also exhibit the antioxidative potential. The analysis of the antiradical activity of chokeberry fruit, juice and pomace against ABTS^•+^ and DPPH^•^ indicated the highest activity of the pomace, followed by the fruit and juice. The antiradical activity was correlated with the polyphenol content in the analysed material [53]. The antioxidative potential of fruit juices was tested. The chokeberry juice had the greatest ability to scavenge the DPPH radical (72.44 µmol TE/mL). The elderberry fruit juice exhibited high activity (62.14 µmol TE/mL), while the activity of other juices was at least 50% lower (4–30 µmol TE/mL) [74]. The ORAC assay confirmed the high antiradical activity of chokeberry juice, which was greater than the activity of 14 other juices [126]. The chokeberry juice concentrate also exhibited the highest antiradical activity, as it scavenged the radicals more effectively than the other fruit juice concentrates [87].

The chokeberry pomace scavenged DPPH radicals and reduced Fe in the FRAP assay much more than the honeysuckle, Japanese quince, blackcurrant and strawberry pomaces. In comparison with the other pomaces the chokeberry pomace exhibited moderate activity in the ABTS^•+^ assay [119]. According to Pieszka et al. [16] dried chokeberry pomace exhibited better antioxidative properties (TRAP) than apple, blackcurrant, strawberry and carrot pomaces. Chokeberry groups differ in their antioxidative activity considerably [45]. Sosnowska et al. [40] studied the antioxidative activity of juices and observed that the results differed about 5 times in ABTS, FRAP and DPPH assays, while chokeberry teas differed 1.1, 1.3, 6.6 and 36.1 times in FRAP, DPPH, reducing power and ABTS assays [108]. There were also differences in the antioxidative activity of dried chokeberry fruits, powders, capsules, and jams [7,34].

The analysis of the results of studies on chokeberry juice shows that the antioxidative potential of chokeberry products depends on the period and the year of raw material harvesting [5,41]. However, the main factor affecting the antioxidative properties of the products that needs to be considered is the technological production processes. The antioxidative activity is influenced by crushed raw material used for the production of juices and chokeberry powders [33], the method and drying parameters during the production of dried fruit and powder extracts [58,88], as well as the extraction solvent and temperature [89,125]. The antioxidative potential of chokeberry fruit and products is mainly attributed to polyphenols. The activity of individual chokeberry polyphenols: anthocyanins, proanthocyanidins, phenolic acids and flavonols was also determined. The major contributor to DPPH radical scavenging values was the anthocyanin fraction (66.7%), followed by the proanthocyanidin fractions (25.1%), flavonols and phenolic acids (8.2% of the total activity) [74,114]. Zheng and Wang [130] assessed the antioxidative activity of chokeberry in the ORAC assay and found that it resulted from anthocyanins (53.1%), phenolic acids (38.2%) and flavonols (8.7%). Proanthocyanidin was not included in the study.

## 5. Conclusions

Black chokeberry (*Aronia melnocarpa*) is a source of many bioactive compounds with a wide spectrum of antioxidant and health-promoting properties. Like other berries, chokeberry is a source of polyphenols, which exhibit the antioxidant potential, demonstrated in numerous in vitro and in vivo experiments. Black chokeberry has the potential to inhibit the activity of various types of radicals, through different mechanisms of action. Fresh, unprocessed black chokeberry fruits are used in the food production, e.g., juices, syrups, wines, preserves, various dietary supplements, and natural dyes. However, they are rarely consumed due to their bitter taste, resulting from the presence of polyphenols. Black chokeberry fruits and flowers are used as traditional remedies based on the health-promoting actions against influenza and immunity enhancer. Numerous studies confirmed the beneficial effects of *Aronia melanocarpa* L. varieties consumption on hypertension, glucose metabolism disorders, dyslipidaemia, proinflammatory conditions, and reducing the risk factors of the metabolic syndrome. Results also showed the probable potential of black chokeberry to inhibit the development of some types of cancers.

Further research is necessary to understand the interactions with other compounds which may affect the activity of chokeberry components. The antioxidant potential of chokeberry fruit and its products indicates that all fractions could be utilized as a source of antioxidants and valuable nutrients with potential applications in food industry. So far the literature has not provided clear answers to questions concerning the mechanism of interaction between chokeberry components and their stability in complex systems, e.g., in food. Studies conducted to date have indicated numerous benefits resulting from chokeberry and its polyphenolic compounds inclusion in a daily diet. Like other plants and medicinal products of natural origin, chokeberry requires extensive studies on humans to determine its efficacy, safety and mechanisms of action.

## Figures and Tables

**Table 1:** The qualitative composition of fruits, juice and other black chokeberry products.

Components	Fruits	Juice	Other Black Chokeberry Products
Dry matter	15.30–19.50% [1], 17.9–26% [11], 26.67–30.76% [13], 15.7% [18], 22.14–23.45% [24], 21.0–26.0% [30], 18.3–23.5 g/100 g FW [31], 18.92–20.14% [32]	19.22–26.94% [5], 11.1–17.4% [11], 15.46–16.87% [33], 13.42–21.54% [34], 18.1% [35]	44.6–50% [11], 90.21% dried pomace [16], 93.6–94.9% dried pomace fractions [19], 96.86% fruit powder [33], 97.63–98.30% pomace powder [33], 90.44–94.797% powder [34], 93.60–93.96% capsules [34], 88.32–96.01% fruit tea [34], 82.00–84.61% dried berries [34], 5.7% extract [36], 90.8% pomace [37], 108 g/kg nectar [38]
Solube solids (ºBrix)	14.20–18.70 [1], 16.0 [12], 21.7–24.1 [13], 8.9–11.2 [17], 15.2–22.9 [23], 15.5–18.2 g/100 g FW [39], 14.4–15.6 [32],	18.15–25.61 [5], 13.30–20.99 [34], 12.50–20.10 [40], 7.8–14.3 [41]	26.75–37.53 powder, 31.91–83.71 capsules, 12.17–38.22 fruit tea, 20.93–25.49 dried berries [34]
Protein	3.71 g/100 g DM [14], 0.60–0.81 g/100g [17], 0.7 g/100g FW [18]	0.2 g/100 g [42,43]	10.77% dried pomace [16], 4.9–24.1% DM dried pomace fractions [19]
Formol number (total amino acid)	10.0–19.9 (ml 0.1 M NaOH/100 g) [23]	-	-
Fat	0.09–0.17 g/100g [17], 0.14% [18]	< 0.1 g/100g [42,43]	5.15% dried pomace [16], 2.9–13.9% DM dried pomace fractions [19], 19.3 g/kg seeds [21]
SFA	-	-	9.51% TFA dried pomace [16]
UFA	-	-	90.49% TFA dried pomace [16]
PUFA *n-6*	-	-	43.60% TFA dried pomace [16]
PUFA *n-3*	-	-	29.97% TFA dried pomace [16]
Sterols	-	-	1.2 g/kg seed oil [21]
Phospholipids	-	-	2.8 g/kg seed oil [21]
Carbohydrates	13.73–15.06 g/100g [17]	17.9 g/100g [42,43]	-
Total dietary fibre	-	0.3 g/100g [42,43]	21.79% dried pomace [16], 63.5–77.9% DM dried pomace fractions [19], 72.0% DM fibre [27], 95.8 g/100 g DM pomace [37]
Soluble fibre	0.81–1.03 g/100 g [17]	-	5.4% DM fibre [27]
Insoluble fibre	4.01–5.25 g/100 g [17]	-	66.6% DM fibre [27]
NDF	-	-	34.65% dried pomace [16], 41.9–48.5% fibre [25], 87.48 g/100 g DM pomace [26]
ADF	-	-	35.59% dried pomace [16], 34.6–45.6% fibre [25], 57.24 g/100 g DM pomace [26]
ADL	-	-	17.58% dried pomace [16]
Lignin	-	-	15.0–26.8% fibre [25], 22.68 g/100 g DM pomace [26], 23.03 g/100 g DM pomace [37]
Cellulose	-	-	18.5–25.1% fibre [25], 34.56 g/100 g DM pomace [26], 33.14 g/100 g DM pomace [37]
Hemicellulose	-	-	0.81–7.72% fibre [25], 30.24 g/100 g DM pomace [26], 32.08 g/100 g DM pomace [37]
Total pectins	-	-	10.7–14.0% fibre [25], 7.52 g/100 g DM pomace [37]
Water soluble pectins	-	-	1.00–2.86% fibre [25]
Total sugars	9.16–13.79 g/100 g [1], 68–158 g/kg [11], 19.32–20.92 g/100 g [13], 83.0–111.6 g/kg [24], 44.596–125.851 g/L [44], 6.21–6.91 g/100 g [32]	110–143 g/L [11], 85.24–87.31 g/100 g DM [33], 12.0–19.6 g/100 mL [45], 89.49–162.37 g/L [40], 14.84% [35]	46.15 g/100 g DM fruit powder [33], 25.05–31.83 g/100 g DM pomace powder [33], 84 g/kg pomace ^11^, 38.2 g/kg nectar [38]
Solube sugars	-	-	0.35% dried pomace [16]
Reducing sugars	8.83–12.48 g/100 g [1], 18.21–19.36 g/100 g [13], 23.00 g/100 g DM [14], 71.5–102.4 g/kg [24], 6.8–9.0 mg/100 g [30], 5.71–6.58 g/100 g [32]	-	-
Sucrose	ND-1.53 g/100 g [13], ND-0.7 g/kg [23], 0.22–0.48 g/100 g [32]	5.4–6.8 g/100 mL [45], ND-3.03 g/L [40], ND [43]	0.03–0.43% DM dried pomace fractions [19], 0.16 g/L extract [36]
Glucose	10.9–40.1 g/kg [11], 36.3–57.0 g/kg [23], 9.794–18.429 g/L [44]	31.7–40.4 g/L [11], 26.15–26.91 g/100 g DM [33], 1.5–3.3 g/100 mL [45], 26.29–42.86 g/L [40], 22.0–34.0 g/kg [41], 4.25 g/100 g [42,43], 3.69% [35]	22.8 g/kg pomace [11], 0.39–0.80% DM dried pomace fractions [19], 14.64 g/100 g DM fruit powder [33], 5.08–12.55 g/100 g DM pomace powder [33], 6.29 g/L extract [36]
Fructose	13.8–41.6 g/kg [11], 26.0–47.1 g/kg [23], 13.828–40.080 g/L [44]	30.2–39.1 g/L [11], 19.04–19.18 g/100 g DM [33], 1.5–3.3 g/100 mL [45], 27.44–41.18 g/L [40], 22.0–32.0 g/kg [41], 3.87 g/100 g [42,43], 2.15% [35]	23.6 g/kg pomace [11], 0.48–0.58% DM dried pomace fractions [19], 10.43 g/100 g DM fruit powder [33], 6.64–6.73 g/100 g DM pomace powder [33], 9.58 g/L extract [36]
Sorbitol	43.6–76.1 g/kg [11], 46.3–85.6 g/kg [23], 20.974–67.342 g/L [44]	47.8–63.8 g/L [11], 39.29–41.99 g/100 g DM [33], 3.6–6.3 g/100 mL [45], 35.77–77.30 g/L [40], 27.4–48.1 g/kg [41], 7.39 g/100 g [42,43], 9.00% [35]	37.6 g/kg pomace [11], 1.06–2.32% DM dried pomace fractions [19],21.08 g/100 g DM fruit powder [33], 12.55–13.33 g/100g DM pomace powder [33], 20.1 g/L extract [36]
pH	3.36–3.79 [17], 3.3–3.7 [18], 3.23–3.57 [31]	3.77–3.96 [5], 3.54–3.92 [34], 3.5 [45], 3.42–3.72 [40], 3.15–3.45 [41], 3.5 [35]	4.02–4.13 powder [34], 3.31–4.10 capsules [34], 4.01–4.13 fruit tea [34], 4.01–4.28 dried berries [34], 3.3 nectar [38]
Titratable acidity (citric acid eq)	0.75–1.05 g/100 g [1], 1.42 g/100 g [12], 0.493–0.548 g/100 g [13], 6.7–11.9 g/kg (eq malic acid) [23], 1.9–2.6 g/kg [24], 1.03–1.44 g/100 g FW (eq malic acid) [31], 1.24–1.31 g/100 g [32]	0.89–1.06% [5], 0.29–1.32% [34], 0.85–1.22% [41], 0.89% [35]	0.52–0.58% DM dried pomace fractions [19], 1.67–2.30% powder [46], 2.10–4.66% capsules [34], 1.08–1.60% fruit tea [34], 1.13–1.37% dried berries [34], 13.2 g/L extract [36], 3.9 g/kg nectar [38]
Organic acids total	8.21–16.812 g/L [44]	12.27–21.87 g/L [40]	-
Citric acid	0.18–0.25 g/100 g [17], 0.7–1.3 g/kg [23], 0.584–2.399 g/L [44]	0.72–2.55 g/L [40]	48.9–94.2 mg/100 g DM dried pomace fractions [19], 10.5 g/L extract [36]
Isocitric acid	17.2–37.3 g/kg [23]	-	-
Malic acid	0.56–1.63 g/100 g [17], 4.5–12.8 g/kg [23], 5.112–11.695 g/L [44]	9.32–13.33 g/L [40]	124.5–301.0 mg/100 g DM dried pomace fractions [19]
Oxalic acid	-	0.17–0.79 g/L [40]	-
Tartaric acid	0.321–2.068 g/L [44]	4.66–5.20 g/L [40]	-
Quinic acid	4.1–6.8 g/kg [23]	-	-
Succinic acid	0.478–0.977 g/L [44]	-	-
Fumaric acid	0.051–0.107 g/L [44]	-	-
Galacturonic acid	-	-	535.4–1561.2 mg/100 g DM dried pomace fractions [19]
Ash	6.83 g/100 g DM [14], 0.37–0.49 g/100g [17], 4.2–11.8 g/kg [23]	0.5 g/100g [42,43]	1.95% dried pomace [16], 1.4–3.9% DM dried pomace fractions [19]
Na	2.0–3.7 mg/100 g [17], 2.6% [18], 12.5–16.8 mg/kg [29]	19.6–56.3 mg/kg [29], 19 ppm [45]	0.037 g/kg dried pomace [16], 52.5–89.0 mg/kg DM dried pomace fractions [19], 9.4–40.8 mg/kg fruit tea [29]
K	164–265 mg/100 g [17], 218 mg% [18], 1356.3–3659.7 mg/kg [23], 2707–4977 mg/kg [29]	848–3204 mg/kg [29], 1242 ppm [45]	2.78 g/kg dried pomace [16], 1814.3–3075.9 mg/kg DM dried pomace fractions [19], 385–2792 mg/kg fruit tea [29]
Ca	22.8–43.9 mg/100 g [17], 32.2 mg% [18], 119.0–552.3 mg/kg [23], 601–1167 mg/kg [29]	138–1225 mg/kg [29], 151 ppm [45]	2.75 g/kg dried pomace [16], 2186.8–4080.4 mg/kg DM dried pomace fractions [19], 469–1395 mg/kg fruit tea [29]
Mg	15.5–17.4 mg/100 g [17], 16.2 mg% [18], 83.3–314.2 mg/kg [23], 164–578 mg/kg [29]	209–589 mg/kg [29], 85 ppm [45]	0.88 g/kg dried pomace [16], 370.8–2501.0 mg/kg DM dried pomace fractions [19], 99–338 mg/kg fruit tea [29]
P	15.9–21.7 mg/100 g [17], 257.0–417.5 mg/kg [23], 239–956 mg/kg [29]	167–1037 mg/kg [29]	2.39 g/kg dried pomace [16], 282–526 mg/kg fruit tea [29]
Zn	0.090–0.220 mg/100 g [17], 4.09–8.40 mg/kg [29]	0.89–3.45 mg/kg [29]	15.7 mg/kg dried pomace [16], 5.6–36.9 mg/kg DM dried pomace fractions [19], 2.41–8.27 mg/kg fruit tea [29]
Fe	0.33–1.68 mg/100 g [17], 0.93 mg% [18], 9.4–14.2 mg/kg [29]	7.2–25.2 mg/kg [29]	197 mg/kg dried pomace [16], 68.9–86.2 mg/kg DM dried pomace fractions [19], 22.8–58.1 mg/kg fruit tea [29]
Se	0.21–0.28 mg/kg [29]	ND-1.73 mg/kg [29]	0.26–0.56 mg/kg fruit tea [29]
Cu	0.035–0.056 mg/100 g [17], 0.82–2.11 mg/kg [29]	0.68–4.51 mg/kg [29]	1.95 mg/kg dried pomace [16], 5.0–12.4 mg/kg DM dried pomace fractions [19], 1.76–4.00 mg/kg fruit tea [29]
Mo	0.016–0.021 mg/kg [29]	ND-0.064 mg/kg [29]	0.050–0.290 mg/kg fruit tea [29]
Mn	0.132–0.263 mg/100 g [17], 5.49–17.89 mg/kg [29]	2.98–11.77 mg/kg [29]	31.5 mg/kg dried pomace [16], 2.63–52.2 mg/kg fruit tea [29]
Ni	0.143–0.740 mg/kg [29]	0.130–0.860 mg/kg [29]	0.204–0.568 mg/kg fruit tea [29]
V	0.40–1.58 mg/kg [29]	0.47–1.43 mg/kg [29]	0.31–0.96 mg/kg fruit tea [29]
Si	2.37–6.37 mg/kg [29]	ND-7.4 mg/kg [29]	2.18–6.30 mg/kg fruit tea [29]
Cr	0.49–0.53 mg/kg [29]	0.55–0.74 mg/kg [29]	0.44–0.85 mg/kg fruit tea [29]
Li	ND-6.75 mg/kg [29]	ND-0.072 mg/kg [29]	ND-0.075 mg/kg fruit tea [29]
Sr	1.57–7.05 mg/kg [29]	0.34–3.67 mg/kg [29]	1.01–9.10 mg/kg fruit tea [29]
Al	2.88–4.40 mg/kg [29]	1.64–9.70 mg/kg [29]	3.60–25.47 mg/kg fruit tea [29]
Sn	0.62–0.72 mg/kg [29]	0.86–1.09 mg/kg [29]	0.58–0.89 mg/kg fruit tea [29]
As	0.20–0.36 mg/kg [29]	0.37–0.79 mg/kg [29]	0.30–0.98 mg/kg fruit tea [29]
Cd	0.016–0.041 mg/kg [29]	0.050–0.064 mg/kg [29]	0.035–0.059 mg/kg fruit tea [29]
Ba	1.48–6.66 mg/kg [29]	0.77–2.06 mg/kg [29]	1.48–9.62 mg/kg fruit tea [29]
Pb	0.048–0.091 mg/kg [29]	ND-0.143 mg/kg [29]	0.053–0.205 mg/kg fruit tea [29]
Sb	ND-0.29 mg/kg [29]	ND-0.54 mg/kg [29]	ND-0.66 mg/kg fruit tea [29]
Co	0.019–0.043 mg/kg [29]	ND-0.092 mg/kg [29]	ND-0.144 mg/kg fruit tea [29]
B	2.88–14.22 mg/kg [29]	ND-9.32 mg/kg [29]	2.60–4.96 mg/kg fruit tea [29]
Vitamin C	31 mg/100 g [12], 1.9–8.4 mg/100 g [13], 4.0–19.3 mg/100 g [17], 13.7 mg/100 g [18], 2.3–13.7 mg/100 g [30], 0.911–1.552 g/L [44], 3.5–7.2 mg/100 g FW [31]	29 g/L [47]	1100 mg/kg nectar [38]
Vitamin B1	0.017–0.019 mg/100 g [17]	-	-
Vitamin B2	0.016–0.027 mg/100 g [17]	-	-
Niacin	0.27–0.34 mg/100 g [17]	-	-
Panthotenic acid	0.225–0.382 mg/100 g [17]	-	-
Vitamin B6	0.024–0.029 mg/100 g [17]	-	-
Folate	0.002–0.004 mg/100 g [17], 20.4–20.6 µg/100 g FW [48]	-	-
Vitamin A	0.77 mg/100 g [18]	-	-
Carotenoids total	48.6 mg/kg [49]	97.8 µg/L [47]	-
Lycopene	ND ^31^, 0.6 mg/kg [49]	-	ND nectar [38]
α-Carotene	6.4–10.6 µg/100 g [17]	-	
β-Carotene	495–887 µg/100 g [17], 46.4 µg/g DW [28], 16.7 mg/kg [49]	-	ND nectar [38]
ζ-Carotene	0.3 mg/kg [49]	-	-
β-Cryptoxanthin	234–649 µg/100 g [17], ND [28], 12.2 mg/kg [49]	-	-
Lutein	9.1 µg/g DW [28], 3.4 mg/kg [49]	-	-
5,6-Epoxylutein	0.4 mg/kg [49]	-	-
*trans*-Violaxanthin	4.5 mg/kg [49]	-	-
*cis*-Violaxanthin	8.5 mg/kg [49]	-	-
Neoxanthin	2.0 mg/kg [49]	-	-
Xanthophyll	2.7 µg/g DW [28]	-	-
Vitamin E		-	55.5 mg/kg seed oil [21]
α-Tocopherol	1.35–1.47 mg/100 g [17]	-	70.6 mg/kg seed oil [21]
β-Tocopherol	0.10–0.16 mg/100 g [17]	-	28.2 mg/kg seed oil [21]
γ-Tocopherol	0.08–0.10 mg/100 g [17]	-	0.2 mg/kg seed oil [21]
γ-Tocotrienol	-	-	0.8 mg/kg seed oil [21]
δ-Tocopherol	0.05–0.07 mg/100 g [17]	-	0.2 mg/kg seed oil [21]
Vitamin K	17.8–28.8 µg/100 g [17]	-	-
Chloride	-	13 ppm [45]	-
Nitrate	45.20–98.50 mg/kg [1], 62.7–64.7 mg/kg [32]	9 ppm [45]	-
Nitrite	0.62–1.87 mg/kg [1], 0.90–1.24 mg/kg [32]	-	-
Phosphate	-	184 ppm [45]	-
Sulfate	-	1368 ppm [45]	-

ADF: acid dietary fibre, ADL: acid detergent lignin, DM: dry matter, FW: fresh weight, NDF: neutral dietary fibre, PUFA: polyunsaturated fatty acids, SFA: saturated fatty acids, TFA: total fatty acids, UFA: unsaturated fatty acids.

**Table 2:** The qualitative composition of selected polyphenols present in black chokeberry fruits, juices and other products.

Compound	Fruit	Juice	Other Products (e.g., Dried Fruits, Extracts, Pomaces, Juice Concentrates, Teas)
Polyphenols total (spectrophotometric method)	1845–2340 mg GAE/100 g [1], 7.78–12.85 g GAE/kg FW [3], 13.3 g GAE/kg [7], 15.0–17.9 g CE/kg FW [11], 603 mg GAE/100 g FW [51], 1079–1921 mg GAE/100 g FW [56], 8008 mg GAE/100 g DM [58], 20.1 mg GAE/g FW [59], 127–197 mg GAE/g DM [67], 1540.01 mg GAE/100 g FW [73], 10637.20 mg GAE/kg [74], 8563.8–12055.7 mg GAE/kg FW [75]	8834–11093 mg GAE/L [5], 6.3–6.6 g GAE/kg [7], 4.7–9.0 g CE/kg FW [11], 3002–6639 mg GAE/L DM [34], 675–755 mg GAE/100 mL [45], 2.73–10.35 g GAE/L [40], 4772.2 mg GAE/L [35], 4.00 g GAE/L [76,77], 8.7 mg GAE/100 g [78], 6484 mg GAE/L [79], 386 mg GAE/100 g [80], 373.5 µg GAE/mL [81], 386 mg GAE/100 g [82], 709.3 mg GAE/100 mL [83], 6652 mg GAE/L [84], 5461 mg GAE/L [85], 3172–7340 mg GAE/L [86]	39.9–50.1 g GAE/kg dried fruits [7], 29.6 g GAE/kg concentrate [7], 63.1 g GAE/kg pomace [7], 6.9–12 g GAE/kg jam [7], 6.7 g GAE/kg compote [7], 2.6 g GAE/kg syrup [7], 31–63 g CE/kg FW pomace [11], 19.64–27.82 mg GAE/g DM extract [57], 4954–7265 mg GAE/100 g DM dried fruits [58], 4233–4951 mg GAE/100 g DM powder [34], 1494–3436 mg GAE/100 g DM fruit tea [34], 1954–2466 mg GAE/100 g DM dried berries [34], 4511–5292 mg GAE/100 g DM capsules [34], 46.8 g GAE/L juice concentrate [87], 27.63–34.28 mg GAE/100 mg DM powder [88], 44.87 mg GAE/g extract [89], 3.38–3.77 g GAE/L juice concentrate [76], 3.68–3.87 g GAE/L juice concentrate [77], 30.9 mg GAE/100 g dried fruit [78], 7.7 mg GAE/100 g compote [78], 792.3–919.7 mg GAE/g DM dried fruits [90], 712 mg GAE/g extract [91], 612.4 mg GA-E/g extract [92], 483 mg CE/g DM extract [93], 757–910 mg GAE/g extract [94], 85.55 mg GAE/100 mL fruit tea (decoction) [95], 88.77 mg GAE/100 mL fruit tea (infusion) [95], 745 mg GAE/g extract [96], 477.72 mg GAE/g extract [97], 714 mg SAE/g extract [98], 2995.20 mg GAE/100 g DM dried fruits [99]
Polyphenols total/sum (chromatographic method)	819.2–1329.5 mg/100 g [1], 672.4 mg/100 g [12], 2574.47–2773.41 mg/100 g FW [13], 7849.21 mg/100 g DM [53], 6351.38 mg/100 g DM [54], 2477.0–6930.5 mg/kg FW [75]	4521.18–6686.69 g/100 g DM [33], 3729.07 mg/100 g DM [53], 996.33–1450 mg/L [40], 1277.09 mg/L [76,77], 6.95 g/L [100], 1296.8–3545.6 µg/mL [101]	8044–15058 mg/100 g DM dried pomace fractions [19], 24723.67 g/100 g DM fruit powder [33], 15607.48–24447.77 g/100 g DM pomace powder [33], 10583.27 mg/100 g DM pomace [53], 63.58 g/kg extract [70], 27.0 g/L juice concentrate [87], 1043.89–1162.77 mg/L juice concentrate [76], 1003.37–1188.62 juice concentrate [77], 0.88 g/L wine [100], 5.29–6.51 g/L juice microcapsules [100], 2.05–2.41 g/L wine microcapsules [100], 42.9–233.9 mg/100 mL liqueur [102], 309.6 mg/g extract [103]
Nonflavonoids total (spectrophotometric method)	-	1383–1840 mg GAE/L [5], 808–1527 mg GAE/L DM [34]	1602–1906 mg GAE/100 g DM powder [34], 2051–2300 mg GAE/100 g DM capsules [34], 479–1557 mg GAE/100 g DM fruit tea [34], 1072–1086 mg GAE/100 g DM dried berries [34]
Phenolic acids total/sum	121.9 mg/100 g [12], 63.9 mg/100 g FW [51], 669.03 mg/100 g DM [54], 183 ng/g DM ^Y^ [104], 32.43 mg/100 g DM [105], 77–96 mg/100 g FW ^Y^ [106]	-	110.92 mg ChAE/g extract [92], 34.5–49.0 mg ChAE/100 mL liqueur [102], 158 ng/g DM compote ^Y^ [104], 74–109 ng/g DM jam ^Y^ [104], 149.2 mg ChAE/g extract [103]
Hydroxycinnamic acids total/sum	1.4–1.5 g ChAE/kg FW [11], 116.4 mg ChAE/100 g FW frozen [63], 127.0 mg ChAE/100 g FW blanched [63], 6.38–9.85 mg/g DM [67], 739.3–1670.3 mg ChAE/kg FW [75]	0.45–0.59 g ChAE/kg FW [11], 48.9–77.9 mg ChAE/100 g FW ^X^ [63]	0.72–0.82 g ChAE/kg FW pomace [11], 89.7–231.6 mg ChAE/100 g DM dried pomace fractions [19], 43.7 mg ChAE/100 g FW presscake ^X^ [63], 0.078 g ChAE/l juice concentrate [87], 271 mg ChAE/100 g juice concentrate [107], 56.7 mg ChAE/g DM extract [93], 135.14 mg ChAE/g extract [97]
Caffeic acid (3,4-Dihydroxycinnamic acid)	0.13 mg/100 g FW ^Z^ [51], ND [62], 3.96 mg/100 g DM ^Z^ [105], 60–75 mg/100 g FW ^Z Y^ [106]	1.2–1.8 mg/L ^Z^ [77], Tr [101]	118.9 µg/100 g herbhoney ^Z^ [69], 0.067–1.26 mg/g tea infusion ^Z^ [108], 0.35 g/L wine [100], 0.6 mg/g extract [91], 0.41–0.48 g/L wine microcapsules [92], 53.85 mg/L extract ^Z^ [109], 0.736 mg/g extract [96], ND dried fruits ^Y^ [99]
Protocatechuic acid (3,4-Dihydroxybenzoic acid)	0.77 mg/100 g FW ^Z^ [51], ND [110], 11 ng/g DM ^Y^ [104], 8.3–13.0 mg/100 g FW ^Z Y^ [106]	24.93–57.35 mg/L ^Z^ [40], 14.3–103.6 µg/mL ^Z^ [101]	ND-0.176 mg/g tea infusion ^Z^ [108],0.08 g/L wine [100], 0.56–0.70 g/L wine microcapsules [100], 2.4 mg/g extract [91], 14 ng/g DM ^Y^ [104], 2–9 ng/g DM ^Y^ [104], 1.94 mg/g extract [96]
Chlorogenic acid (3-Caffeoylquinic acid) 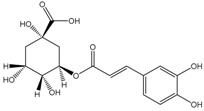	72.0–96.6 mg/100 g [1], 1131.15–1960.72 mg/kg FW [3], 0.69–0.74 g/kg FW ^Z^ [11], 65.42 mg/100 g [12], 83.97–110.62 mg/100 g FW ^Z^ [13], 20.4 mg NChaE/100 g FW [51], 301.85 mg/100 g DM ^Z^ [53], 164.42 mg/100 g FW ^Z^ [62], 70.2 mg/100 g FW frozen ^Z^ [63], 80.2 mg/100 g FW blanched ^Z^ [63], 3.32–6.42 mg/g DM ^Z^ [67], 218 mg/100 g FW ^Z^ [110], 61 mg/100 g FW ^Z^ [72], 416.9–1000.6 mg/kg FW ^Z^ [75]	0.2–0.3 g/kg FW ^Z^ [11], 470.51–642.74 mg/100 g DM ^Z^ [33], 415.86 mg/100 g DM ^Z^ [53] 30.4–47.7 mg/100 g FW ^Z X^ [63], 58.9–67.8 mg/100 mL [45], 463.31–642.28 mg NChAE/l [40], 390.5 mg/L ^Z^ [35], 10.3–36.3 mg/L ^Z^ [111] 370.06 mg/L ^Z^ [76,77], 0.51 mg/g ^Z^ [78], 1389 mg/L ^Z^ [79], 0.97 g/L [100], 32 mg/100 g [82], 691 mg/L [84], 858 mg/L [85], 453.3–628.1 µg/mL ^Z^ [101], 45.50 mg/100 mL ^Z^ [112]	0.42–0.50 g/kg FW pomace ^Z^ [11],33.2–84.5 mg/100 g DM dried pomace fractions ^Z^ [19], 769.25 mg/100 g DM fruit powder ^Z^ [33], 848.17–1192.69 mg/100 g DM dried pomace powder ^Z^ [33], 204.35 mg/100 g DM pomace ^Z^ [53], 3.60–4.60 mg/g DM dried fruits [54], 28.8 mg/100 g FW presscake ^Z X^ [63], 13.7 µg/100 g herbhoney ^Z^ [69], 6.24 g/kg extract [70], 43.95 mg NChAE/g DM extract [113], 0.0377 g/L juice concentrate ^Z^ [87], 603.1 mg/kg extract ^Z^ [114], 310.59–354.07 mg/L juice concentrate ^Z^ [76], 318.19–352.34 mg/L juice concentrate ^Z^ [77], 2.33 mg/g dried fruit ^Z^ [78], 0.37 mg/g compote ^Z^ [78], 20.6 μg/mg extract ^Z^ [115], 0.94–0.99 g/L juice microcapsules [100], 63.8 mg/g extract [91], 68.32 mg/g extract ^Z^ [92], 2181.05 mg/L extract [109], 79.0 mg/g extract [96], 3.90 mg/g extract ^Z^ [97], 63.5 mg/g extract [98], 58.23 mg/100 g DM dried fruits ^Z Y^ [99]
Neochlorogenic acid (5-Caffeoylquinic acid) 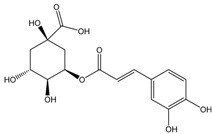	59.3–79.1 mg/100 g [1], 883.31–1156.59 mg/kg FW [3], 0.71–0.72 g ChAE/kg FW [11], 56.51 mg/100 g [12], 74.60–99.76 mg ChAE/100 g FW [13], 37.5 mg/100 g FW ^Z^ [51], 290.81 mg ChAE/100 g DM [53], 79.88 mg/100 g FW ^Z^ [62], 46.2 mg ChAE/100 g FW frozen [63], 46.8 mg ChAE/100 g FW blanched [63], 2.16–6.54 mg ChAE/g DM [67], 123 mg ChAE/100 g FW [72], 189 mg ChAE/100 g FW [110], 322.4–669.7 mg ChAE/kg FW [75]	0.21–0.29 g ChAE/kg FW [11], 891.56–1048.49 mg/100 g DM [33], 393.10 mg ChAE/100 g DM [53], 18.4–30.1 mg ChAE/100 g FW ^X^ [63], 65.5–80.6 mg/100 mL [45], 323.67–442.33 mg/L ^Z^ [40], 415.7 mg/L ^Z^ [35], 21.8–57.3 mg/L [111], 426.57 mg ChAE/l [76,77], 0.47 mg ChAE/g [78], 1057 mg/L ^Z^ [79], 1.18 g/L [100], 28 mg/100 g [82], 840 mg/L [84], 830 mg/L [85], 361.3–449.0 µg ChAE/mL [101], 49.21 mg ChAE/100 mL [112]	25.5–68.1 mg ChAE/100 g DM dried pomace fractions [19], 728.81 mg/100 g DM fruit powder [33], 1161.53–1174.35 mg/100 g DM pomace powder [33], 169.20 mg ChAE/100 g DM pomace [53], 14.9 mg ChAE/100 g FW presscake ^X^ [63], 6.03 g/kg extract [70], 1.13 mg/g DM extract ^Z^ [113], 0.0401 g ChAE/l juice concentrate [87], 257.2 mg/kg extract ^Z^ [114], 355.51–402.08 mg ChAE/l juice concentrate [76], 334.52–422.32 mg ChAE/l juice concentrate [77], 1.82 mg ChAE/g dried fruit [78], 0.26 mg ChAE/g compote [78], 1.25–1.28 g/L juice microcapsules [100], 7 mg/g extract [91], 21.6 mg/g extract [96], 0.31–0.32 g ChAE/kg FW pomace [96], 24.05 mg ChAE/g extract [97], 41.7 mg/g extract [98]
Cryptochlorogenic acid (4-Caffeoylquinic acid)	2.22 mg NChAE/100 g FW [51], ND [62]	28.21–32.66 mg/100 g DM [33], 34.56–86.51 mg NChAE/l [40], 0.13 mg ChAE/g [78]	19.91 mg/100 g DM fruit powder [33], 41.57–53.60 mg/100 g DM pomace powder [33], ], ND extract [113], 0.17 mg ChAE/g dried fruit [79], 0.03 mg ChAE/g compote [79]
Gallic acid (3,4,5-Trihydroxybenzoic acid)	ND [51], ND ^Y^ [106]	0.04 g/L [100], 6.9 mg/L [84], ND-197.8 µg/mL ^Z^ [101]	111.3 µg/100 g herbhoney ^Z^ [69], ND-0.596 mg/g tea infusion ^Z^ [108], 3.15 mg/100 g DM dried fruits ^Z Y^ [99]
Vanillic acid (3-Methoxy-4-hydroxybenzoic acid)	0.37 mg/100 g DM ^Z^ [105], ND-1.31 mg/100 g FW ^Z Y^ [106]	-	0.07–0.09 mg/g DM dried fruits [90], ND dried fruits ^Y^ [99]
*p*-Coumaric acid (4-hydroxycinnamic acid)	ND [12], 0.02 mg/100 g FW ^Z^ [51], ND [62], 27 ng/g DM ^Z Y^ [104], 3.05 mg/100 g DM ^Z^ [105], 5.5–7.61 mg/100 g FW ^Z Y^ [106]	-	446.4 µg/100 g herbhoney ^Z^ [69], ND extract [113], 4.3 μg/mg extract ^Z^ [115], 24 ng/g DM compote ^Z Y^ [104], 12–25 ng/g DM jam ^Z Y^ [104], 7.33 mg/100 g DM dried fruits ^Z Y^ [99]
Cinnamic acid	ND-0.90 mg/100 g FW ^Z Y^ [106]	-	7.5 μg/mg extract ^Z^ [115]
*p*-Coumaric acid derivatives	-	0.4 mg ChAE/100 mL [112]	-
Coumaric acid glucoside	1.29 mg CoAE/100 g FW [51]	-	-
Caffeoylquinic acid derivative	-	19.3–128.3 µg ChAE/mL [101]	-
Caffeic acid glucoside	0.04 mg CaE/100 g FW [51]	-	-
*p*-Coumaroylquinic acid 3-*O*-*p*-Coumaroylquinic acid	-	8.31–9.32 mg/100 g DM [33], 2.72–3.66 mg CoAE/l [40]	6.81 mg/100 g DM fruit powder [33], 10.96–12.70 mg/100 g DM pomace powder [33], ND extract [113]
dicaffeoylquinic acid	3.74 mg/100 g FW [62]	-	0.26 mg NChAE/g DM extract [113]
di-Caffeic quinic acid	-	1.00–1.33 mg/100 g DM [33]	4.88 mg/100 g DM fruit powder [33], 3.09–5.35 mg/100 g DM pomace powder [33]
Ferulic acid (3-(4-Hydroxy-3-methoxyphenyl)-2-propenoic acid)	0.01 mg/100 g FW ^Z^ [51], 1.9–2.8 mg/100 g FW ^Z Y^ [106]	19.9 mg/L [84]	41.4 µg/100 g herbhoney ^Z^ [69], 15.09 mg/L extract [109], ND dried fruits ^Y^ [99]
Depside	-	2.15–6.30 PCAE mg/L [40]	-
Gentisic acid (2,5-Dihydroxybenzoic acid)	-	-	229.2 µg/100 g herbhoney ^Z^ [69]
Sinapic acid (3,5-Dimethoxy-4-hydroxycinnamic acid)	ND ^Y^ [106]	-	44.6 µg/100 g herbhoney ^Z^ [69]
Salicylic acid (*o*-Hydroxybenzoic acid)	15.60 mg/100 g DM ^Z^ [67]	-	-
Syringic acid (4-Hydroxy-3,5-dimethoxybenzoic acid)	65 ng/g DM ^Y^ [104], 4.16 mg/100 g DM ^Z^ [105], ND ^Y^ [106]	-	7.8 µg/100 g herbhoney ^Z^ [69], 53 ng/g DM compote ^Y^ [104], 34–45 ng/g DM jam ^Y^ [104]
4-Hydroxybenzoic acid	ND [51], 38 ng/g DM ^Y^ [104], 5.29 mg/100 g DM ^Z^ [105], ND ^Y^ [106]	-	35 ng/g DM compote ^Y^ [104], 9–26 ng/g DM jam ^Y^ [104], 13 ng/g DM dried homogenate prepared from fresh fruit ^Y^ [104]
Ellagic acid	1.57 mg/100 g FW ^Z^ [51], 42 ng/g DM ^Y^ [104]	-	ND extract [71], ND extract [113], 32 ng/g DM compote ^Y^ [104], 11–15 ng/g DM jam ^Y^ [104], ND dried fruits ^Y^ [99]
Flavonoids total (spectrophotometric method)	5.3 g CE/kg [7], 18.31 mg QE/100 g FW [73]	6994–9710 mg GAE/L [5], 2.7–2.9 g CE/kg [7], 2180–5271 mg GAE/L DM [34], 2.50 g CE/l [76,77], 189.4 mg CE/100 mL [83]	12.5–19.9 g CE/kg dried fruits [7], 6.1 g CE/kg concentrate [7], 9.3 g CE/kg pomace [7], 2.9–6.4 g CE/kg jam [7], 3.3 g CE/kg compote [7], 1 g CE/kg syrup [7], 2327–3317 mg GAE/100 g DM powder [34], 2459–2992 mg GAE/100 g DM capsules [34], 878–2322 mg GAE/100 g DM fruit tea [34], 867–1394 mg GAE/100 g DM dried berries [34], 3.45–5.22 mg QE/100 mg DM powder [88], 5.65 mg CE/g extract [89], 2.01–2.27 g CE/l juice concentrate [76], 1.91–2.27 g CE/l juice concentrate [77], 52.0–66.1 mg CE/g DM dried fruits [90], 21.94 mg/g extract [92], 62.0–90.1 mg CE/g extract [94]
Flavonoids total (chromatographic method)	556.0 mg/100 g [12], 71 mg QRE/100 g FW [72]	-	49.7 mg QRE/g extract [103]
Anthocyanins total (spectrophotometric method)	4.5 g CGlE/kg [7], 488.8 mg CGlE/100 g FW frozen [8], 3917 mg CGlE/100 g DM [58], 498.98 mg/100 g FW [73], 4341.06 mg CGlE/kg [74]	1829–2768 mg CGlE/l [5], 130.5–210.3 mg CGlE/100 g FW ^X^ [8], 150–1228 mg CGlE/l DM [34], 0.10–0.67 g CGlE/l [40], 0.4–0.7 g CGlE/kg [41], 456.2 CGlE/l [35], 369.47 mg CGlE/l [76,77], 0.6 CGlE mg/100 g [78], 106.8 mg CGlE/100 mL [83], 508–1087 mg CGaE/l [86]	1.4–3.1 g CGlE/kg dried fruits [7], 3.6 g CGlE/kg concentrate [7], 10 g CGlE/kg pomace [7], 0.2–0.4 g CGlE/kg jam [7], 0.2 g CGlE/kg compote [7], 0.1 g CGlE/kg syrup [7], 515.6–652.9 mg CGlE/100 g FW of mash [8], 1581.7–2495.2 mg CGlE/100 g FW of pomace [8], 238.5–383.6 mg CGlE/100 g FW of pomace ^X^ [8], 781–2227 mg CGlE/100 g DM dried fruits [58], 1165–1641 mg CGlE/100 g DM powder [34], 1997–2468 mg CGlE/100 g DM capsules [34], 282–675 mg CGlE/100 g DM fruit tea [34], 141–147 mg CGlE/100 g DM dried berries [34], 2.93–4.80 mg CGlE/100 mg DM powder [88], 0.203–0.273 CGlE% DM extract [111], 271.35–330.32 g CGlE/l juice concentrate [76], 270.10–352.30 g CGlE/l juice concentrate [77], 7.1 CGlE mg/100 g dried fruit [79], 0.8 CGlE mg/100 g compote [78], 1146–3715 mg CGlE/g DM dried fruits [90], 202.28 mg/g extract [92], 0.07–0.14 mg CGlE/100 g extract [94], 8.12 mg CGlE/100 mL fruit tea (decoction) [95], 8.63 mg CGlE/100 mL fruit tea (infusion) [95]
Anthocyanins total/sum (chromatographic method)	256.4 mg/100 g FW frozen [8], 6.2–6.7 g CGlE/kg FW [11], 529.3 mg/100 g [12], 357 mg CGlE/100 g FW [51], 1265.48 mg CRE/100 g DM [54], 249–447 mg CGaE/100 g FW [56], 1480.0 mg CGlE/kg [59], 619.2 mg CGlE/100 g FW frozen [63], 281.2 mg CGlE/100 g FW blanched [63], 3.37–14.87 mg CGaE/g DM [67], 4056.22 mg CGlE/kg [74], 1500.9–5486.2 mg CGlE/kg FW [75]	63.9–98.7 mg/100 g FW ^X^ [8], 58–473 g CGlE/kg FW [11], 7.2–104.8 mg CGlE/100 g FW ^X^ [63], 2.8–45.2 mg CGaE/100 mL [45], 373.06 mg/L [76,77], 4.76 g/L [100], 59.3–1118 mg CGlE/l [116], 221.4 mg/L [85], 2.0–855.5 µg CGlE/mL [101], 19.10 mg/100 mL [117]	274.5–310.6 mg/100 g FW mash [8], 738.7–1221.1 mg/100 g FW pomace [8], 114.4–186.0 mg/100 g FW pomace ^X^ [8], 11.9–19.5 g CGlE/kg FW pomace [11], 616.2–1239.0 mg CGlE/100 g DM dried pomace fractions [19], 138.3 mg CGlE/100 g FW presscake ^X^ [63], 8.0 g CRE/l juice concentrate [87], 8384 mg CGlE/kg extract [114], 957.2 mg/100 g juice concentrate [107], 238.35–317.02 mg/L juice concentrate [76], 258.84–316.50 mg/L juice concentrate [77], 0.45 g/L wine [100], 3.07–4.27 g/L juice microcapsules [100], 1.02–1.30 g/L wine microcapsules [100], 147 mg CGlE/g DM extract [93], 5.9 mg CGlE/g extract capsule [116], 188 mg CGlE/l syrup [116], 1.2–170.7 mg CGlE/100 mL liqueur [102], 93.75 mg CGlE/g extract [97], 110.7 mg CGaE/g extract [103], 23.6–192.1 mg/L dried fruits infusions [118], 272.2–342.1 mg/L dried pomace infusions [118]
Cyanidin-3-*O*-arabinoside 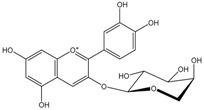	128.0–299.4 mg/100 g [1], 941.82–1553.29 mg/kg FW [3], 1359.4 mg CGlE/kg [7], 74.3 mg/100 g FW frozen ^Z^ [8], 1.9–2.1 g CGlE/kg FW [11], 116.39 mg/100 g [12], 220.27–249.46 mg CGaE/100 g FW [13], 112 mg CGlE/100 g FW [51], 581.50 mg CGaE/100 g DM [53], 52–149 mg CGaE/100 g FW [56], 399.3 mg CGlE/100 g FW [59], 1243.2 mg CyE/kg FW [60], 159.21 mg/100 g FW ^Z^ [62], 154.7 mg CGlE/100 g FW frozen [63], 57.5 mg CGlE/100 g FW blanched [63], 0.18–4.06 mg CGaE/g DM [67], 146 mg CGaE/100 g FW [72], 544 mg CGlE/100 g FW [110], 993.77 mg CGlE/kg [74], 367.2–1532.4 mg CGlE/kg FW [75]	117.8–172.6 mg CGlE/kg [7], 16.9–27.7 mg/100 g FW ^Z X^ [8], 14–108 g CGlE/kg FW [11], 248.72–554.90 mg/100 g DM [33], 324.37 mg CGaE/100 g DM [53], 1.0–20.4 mg CGlE/100 g FW ^Z^ [63], 0.7–10.7 mg CGaE/100 mL [45], 11.47–32.59 CGlE mg/L [40], 28.8–48.5 mg/L ^Z^ [111], 78.47 mg/L [76,77], 101 mg/L ^Z^ [79], 1.44 g/L [100], 36.2 mg CGaE/100 g [80], 1.93 mg/100 mL ^Z^ [81], 11.3–249.9 mg CGlE/l [116], 36.2 mg/100 g [82], 8.2 mg/L [84], 61.7 mg/L [85], Tr-190.2 µg CGlE/mL [101], 5.18 mg/100 mL ^Z^ [117], 110.1–178.7 mg CGaE/l [86], 5.12 mg CGaE/100 mL [112]	186–477.7 mg CGlE/kg dried fruits [7], 1447.6 mg CGlE/kg concentrate [7], 1651.1 mg CGlE/kg pomace [7], 22–85.2 mg CGlE/kg jam [7], 41 mg CGlE/kg compote [7], 27.2 mg CGlE/kg syrup [7], 84.2–93.4 mg/100 g FW mash ^Z^ [8], 217.5–366.7 mg/100 g FW pomace ^Z^ [8], 36.0–55.8 mg/100 g FW pomace ^Z X^ [8], 3.7–5.7 g CGlE/kg FW pomace [11], 23.38 mg CGlE/g extract [11], 191.7–389.9 mg CGlE/100 g DM dried pomace fractions [19], 3328.79 mg/100 g DM fruit powder [33], 1835.62–3116.02 mg/100 g DM pomace powder [33], 532.64 mg CGaE/100 g DM pomace [53], 0.14–0.32 mg/g DM extract ^Z^ [57], 29.3 mg CGlE/100 g FW presscake ^Z^ [63], 6.17 g/kg extract [70], 77.08 mg CGlE/g DM extract [113], 0.0274 g CRE/l juice concentrate [87],1.9–17.7 mg/g tea infusion ^Z^ [108], 2143.0 mg CGlE/kg extract [114], 187.9 mg/100 g juice concentrate ^Z^ [107], 45.19–64.55 mg/L juice concentrate [76], 49.76–67.77 mg/L juice concentrate [77], 51.6–370.9 mg/g DM dried fruits [90], 159.6 μg CGlE/mg extract [115], 0.25 g/L wine [100], 1.03–1.26 g/L juice microcapsules [100], 0.46–0.59 g/L wine microcapsules [100], 23.58 mg/g extract [91], 33.21 mg/g extract ^Z^ [92], 1.42 mg CGlE/g extract capsule [116], 36.3 mg CGlE/l syrup [116], 2.43 mg/100 mL fruit tea (decoction) ^Z^ [95], 2.32 mg/100 mL fruit tea (infusion) ^Z^ [95], 36.7 mg CGaE/g extract [96], 81.8 mg/g extract [98], 5.9–54.6 mg/L dried fruits infusions [118], 77.3–98.7 mg/L dried pomace infusions [118]
Cyanidin-3-*O*-galactoside (Idaein) 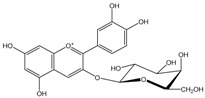	417.3–636.0 mg/100 g [1], 1010.80–1203.56 mg/kg FW [3], 2917.2 mg CGlE/kg [7], 157.1 mg/100 g FW frozen ^Z^ [8], 4.1–4.4 g CGlE/kg FW [11], 379.36 mg/100 g [12], 473.54–515.22 mg/100 g FW ^Z^ [13], 229 mg CGlE/100 g FW [51], 1282.41 mg/100 g DM ^Z^ [53],168–282 mg/100 g FW ^Z^ [56], 989.7 mg CGlE/100 g FW [59], 2482.4 mg/kg FW ^Z^ [60], 222.11 mg/100 g FW ^Z^ [62], 424.7 mg CGlE/100 g FW frozen [63], 205.5 mg CGlE/100 g FW blanched [63], 2.21–14.50 mg/g DM ^Z^ [67], 315 mg CGaE/100 g FW ^Z^ [72], 1243 mg CGlE/100 g FW [110], 2794.74 mg CGlE/kg [74], 1055.3–3621.0 mg CGlE/kg FW [75]	286.6–441.4 mg CGlE/kg [7], 40.1–60.3 mg/100 g FW ^Z X^ [8], 43–341 g CGlE/kg FW [11], 702.15–1451.55 mg/100 g DM ^Z^ [33], 787.00 mg/100 g DM ^Z^ [53], 5.5–77.1 mg CGlE/100 g FW ^X^ [63], 1.7–29.5 mg/100 mL ^Z^ [45], 46.58–96.88 CGlE mg/L [40], 82.1–133.0 mg/L ^Z^ [111], 278.43 mg/L ^Z^ [76,77], 301 mg CArE/l [79], 3.16 g/L [100], 107.6 mg/100 g ^Z^ [80], 5.48 mg/100 mL ^Z^ [81], 44.0–822.1 mg CGlE/l [116], 107.6 mg/100 g [82], 20.0 mg/L [84], 143.7 mg/L [85], 1.9–616.0 µg CGlE/mL [101], 12.60 mg/100 mL ^Z^ [117], 319.4–506.1 mg/L ^Z^ [86], 12.49 mg/100 mL ^Z^ [112]	475.7–928 mg CGlE/kg dried fruits [7], 3349.7 mg CGlE/kg concentrate [7], 4600.5 mg CGlE/kg pomace [7], 81.2–237.4 mg CGlE/kg jam [7], 120.4 mg CGlE/kg compote [7], 81.6 mg CGlE/kg syrup [7], 162.0–187.4 mg/100 g FW mash ^Z^ [8], 437.2–754.6 mg/100 g FW pomace ^Z^ [8], 68.4–114.9 mg/100 g FW pomace ^Z X^ [8], 7.6–12.5 g CGlE/kg FW pomace [11], 376.5–749.4 mg CGlE/100 g DM dried pomace fractions [19], 8286.4 mg/100 g DM fruit powder ^Z^ [33], 4521.34–7961.70 mg/100 g DM pomace powder ^Z^ [33], 1119.70 mg/100 g DM pomace ^Z^ [53], 0.40–0.85 mg/g DM extract ^Z^ [57], 99.8 mg CGlE/100 g FW presscake ^X^ [63], 15.53 g/kg extract [70], 181.01 mg CGlE/g DM extract [113], 0.0432 g CRE/l juice concentrate [87], 3.8–37.6 mg/g tea infusion ^Z^ [108], 5456.0 mg CGlE/kg extract [114], 733.3 mg/100 g juice concentrate ^Z^ [107], 182.32–238.69 mg/L juice concentrate ^Z^ [76], 196.63–240.36 mg/L juice concentrate ^Z^ [77], 119.6–798.1 mg/g DM dried fruits [90], 314.0 μg CGlE/mg extract [115], 0.20 g/L wine [100], 1.99–2.81 g/L juice microcapsules [100], 0.56–0.71 g/L wine microcapsules [100], 80.07 mg/g extract ^Z^ [92], 4.03 mg CGlE/g extract capsule [116], 144.1 mg CGlE/l syrup [116], 6.66 mg/100 mL fruit tea (decoction) ^Z^ [95], 5.04 mg/100 mL fruit tea (infusion) ^Z^ [95], 84.5 mg/g extract ^Z^ [96], 64.04 mg CGlE/g extract [97], 270.2 mg/g extract [98], 16.0–126.2 mg/L dried fruits infusions [118], 178.2–222.7 mg/L dried pomace infusions [118]
Cyanidin 3-*O*-glucoside (Chrysanthemin) 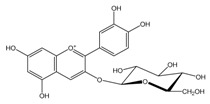	7.8–27.2 mg/100 g [1], 127 mg/kg ^Z^ [7], 11.1 mg/100 g FW frozen ^Z^ [8], 0.08–0.09 g/kg FW ^Z^ [11], 7.11 mg/100 g [12], 18.15–21.51 mg/100 g FW ^Z^ [13], 7.66 mg/100 g FW ^Z^ [51], 42.14 mg CGaE/100 g DM [53], Tr-4.7 mg CGaE/100 g FW [56], 37.6 mg/100 g FW ^Z^ [59], 200.0 mg/kg FW ^Z^ [60], 10.87 mg/100 g FW ^Z^ [62], 19.8 mg/100 g FW frozen ^Z^ [63], 10.9 mg/100 g FW blanched ^Z^ [63], 0.049–0.469 mg CGaE/g DM [67], 10 mg CGaE/100 g FW [72], 46.2 mg/100 g FW ^Z^ [110], 121.69 mg/kg ^Z^ [74], 34.1–113.5 mg/kg FW ^Z^ [75]	15.2–19.9 mg/kg ^Z^ [7], 2.9–4.8 mg/100 g FW ^Z X^ [8], 0.5–9.9 g/kg FW ^Z^ [11], 19.71–39.99 mg/100 g DM [33], 28.15 mg CGaE/100 g DM [53], 0.4–4.6 mg/100 g FW ^Z X^ [63], 0.3–2.0 mg CGaE/100 mL [45], 2.01–4.37 mg/L ^Z^ [40], 3.7–5.7 mg/L ^Z^ [111], 9.28 mg/L ^Z^ [76,77], 21 mg/L ^Z^ [79], 0.16 g/L [100], 0.72 mg/100 mL ^Z^ [81], 2.4–41.9 mg/L ^Z^ [116], 4.9 mg/100 g [82], 4.4 mg/L [84], 4.4 mg/L [85], Tr-25.1 µg/mL ^Z^ [101], 0.73 mg/100 mL ^Z^ [117], 24.0–43.7 mg CGaE/l [86], 0.71 CGaE/100 mL [112]	19.3–60.6 mg/kg dried fruits ^Z^ [7], 214.7 mg/kg concentrate ^Z^ [7], 237.7 mg/kg pomace ^Z^ [7], 3.3–10 mg/kg jam ^Z^ [7], 4 mg/kg compote ^Z^ [7], 3.6 mg/kg syrup ^Z^ [7],12.2–13.3 mg/100 g FW mash ^Z^ [8], 33.9–52.0 mg/100 g FW pomace ^Z^ [8], 5.1–7.9 mg/100 g FW pomace ^Z X^ [8], 0.24–0.44 g/kg FW pomace ^Z^ [11], 21.0–43.7 mg/100 g DM dried pomace fractions ^Z^ [19], 5.4 mg/100 g FW presscake ^Z X^ [63], 225.80 mg/100 g DM fruit powder [33], 125.91–220.06 mg/100 g DM pomace powder [33], 79.44 mg CGaE/100 g DM pomace [53], 0.07–0.14 mg/g DM extract ^Z^ [57], 0.79 g/kg extract [70], 7.09 mg/g DM extract ^Z^ [113], 0.0055 g CRE/l juice concentrate [87], 0.52–5.6 mg/g tea infusion ^Z^ [108], 13.84–21.10 mg/100 mg DM powder ^Z^ [88], 415.0 mg/kg extract ^Z^ [114], 34.1 mg/100 g juice concentrate ^Z^ [107], 6.52–7.52 mg/L juice concentrate ^Z^ [76], 6.87–8.15 mg/L juice concentrate ^Z^ [77], 6.1–49.1 mg/g DM dried fruits [90], 14.5 μg/mg extract ^Z^ [115], 0.05–0.20 g/L juice microcapsules [100], 3.68 mg/g extract ^Z^ [92], 0.196 mg/g extract capsule ^Z^ [116], 7.5 mg/L syrup ^Z^ [116], 1.29 mg/100 mL fruit tea (decoction) ^Z^ [95], 0.85 mg/100 mL fruit tea (infusion) ^Z^ [95], 4.79 mg CGaE/g extract [96], 3.14 mg/g extract ^Z^ [97], 12.5 mg/g extract [98], 0.7–4.3 mg/L dried fruits infusions [118], 6.9–8.0 mg/L dried pomace infusions [118]
Cyanidin-3-*O*-xyloside 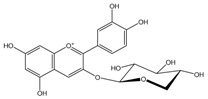	29.0–38.2 mg/100 g [1], 165.8 mg CGlE/kg [7], 13.7 mg CGlE/100 g FW frozen [8], 0.13–0.14 g CGlE/kg FW [11], 26.40 mg/100 g [12], 30.27–33.39 mg CGaE/100 g FW [13], 8.12 mg CGlE/100 g FW [51], Tr-12 mg CGaE/100 g FW [56], 52.71 mg CGaE/100 g DM [53], 51.5 mg CGlE/100 g FW [59], 38.7 mg CyE/kg FW [60], 9.92 mg/100 g FW [62], 20.1 mg CGlE/100 g FW frozen [63], 7.3 mg CGlE/100 g FW blanched [63], ND-0.391 mg CGaE/g DM [67], 10 mg CGaE/100 g FW [72], 73 mg CGlE/100 g FW [110], 146.02 mg CGlE/kg [74], 44.3–233.1 mg CGlE/kg FW [75]	14.7–19.3 mg CGlE/kg [7], 3.7–5.8 mg CGlE/100 g FW ^X^ [8], 1–13 g CGlE/kg FW [11], 17.32–48.35 mg/100 g DM [33], 33.63 mg CGaE/100 g DM [53], 0.21–2.7 mg CGlE/100 g FW ^X^ [63], 1.24–4.74 CGlE mg/L [40], 0.2–2.1 mg CGaE/100 mL [45], 3.2–5.2 mg/L [111], 6.88 mg/L [76,77], 13 mg CArE/l [79], 5.2 mg CGaE/100 g [80], 0.8–3.8 mg CGlE/l [116], 5.2 mg/100 g [82], 0.6 mg/L [84], 11.6 mg/L [85], Tr-24.3 µg CGlE/mL [101], 0.59 mg/100 mL ^Z^ [117], 19.8–29.4 mg CGaE/l [86], 0.59 CGaE/100 mL [112]	21.8–62.5 mg CGlE/kg dried fruits [7], 201.1 mg CGlE/kg concentrate [7], 223.4 mg CGlE/kg pomace [7], 3–8.7 mg CGlE/kg jam [7], ND mg CGlE/kg compote [7], 2.8 mg CGlE/kg syrup [7], 15.8–17.2 mg CGlE/100 g FW mash [8], 36.7–63.3 mg CGlE/100 g FW pomace [8], 4.7–7.3 mg CGlE/100 g FW pomace ^X^ [8], 0.3–0.6 g CGlE/kg FW pomace [11], 3.19 mg CGlE/g extract [11], 27.0–57.1 mg CGlE/100 g DM dried pomace fractions [19], 294.14 mg/100 g DM fruit powder [33], 166.86–275.41 mg/100 g DM pomace powder [33], 105.06 mg CGaE/100 g DM pomace [53], 3.7 mg CGlE/100 g FW presscake ^X^ [63], 1.03 g/kg extract [70], 8.17 mg CGlE/g DM extract [113], 0.0031 g CRE/l juice concentrate [87], 0.77–4.4 mg CGlE/g tea infusion [108], 334.0 mg CGlE/kg extract [114], 4.32–6.43 mg/L juice concentrate [76], 4.70–6.55 mg/L juice concentrate [77], 5.2–71.2 mg/g DM dried fruits [90], 16.6 μg CGlE/mg extract [115], 1.52 mg/g extract [91], 0.255 mg CGlE/g extract capsule [116], 0.45 mg CGlE/l syrup [116], 0.96 mg CGlE/100 mL fruit tea (decoction) [95], 0.99 mg CGlE/100 mL fruit tea (infusion) [95], 5.05 mg CGaE/g extract [96], 40.0 mg/g extract [98], 0.7–7.0 mg/L dried fruits infusions [118], 10.1–12.7 mg/L dried pomace infusions [118]
Cyanidin-3-*O*-galactoside + Cyanidin-3-O-glucoside	-	-	65.04 mg/g extract [91]
Cyanidin-3,5-hexoside-(epi)catechin	-	9.16–9.87 mg/100 g DM [33]	12.04 mg/100 g DM fruit powder [33], 14.33–20.43 mg/100 g DM pomace powder [33]
Cyanidin-3-pentoside-(epi)catechin	-	3.95–4.24 mg/100 g DM [33]	5.76 mg/100 g DM fruit powder [33], 7.26–10.30 mg/100 g DM pomace powder [33]
Cyanidin-3-hexoside-(epi)cat-(epi)cat	-	6.77–7.74 mg/100 g DM [33]	10.98 mg/100 g DM fruit powder [33], 13.61–20.23 mg/100 g DM pomace powder [33]
Cyanidin	-	0.22 mg CGaE/100 mL [112]	387.43 mg CyE/100 g DM dried fruits ^Y^ [99]
Pelargonidin-3-arabinoside	2.3 mg CGlE/100 g FW [59], 50.4 mg PE/kg FW [60]	-	7.6 mg/100 g juice concentrate ^Z^ [107]
Pelargonidin-3-arabinoside + Pelargonidin-3-galactoside	-	-	0.473 mg CGaE/g extract [96]
Pelargonidin-3-galactoside	Tr [59], ND [60]	-	-
Flavanones			
Eriodictyol-7-glucuronide Eriodictyol-glucuronide	22.11–26.43 mg/100 g FW [13]	19.24–28.97 mg/100 g DM [33], 24.31–64.88 NE mg/L [40]	81.36 mg/100 g DM fruit powder [33], 57.61–84.40 mg/100 g DM pomace powder [33]
Eriodictyol-3,7-*O*-diglucuronide	-	7.86 mg/100 mL [112]	1.86 g/kg extract [70]
Eriodictyol	51.4 mg/100 g FW [119]	-	-
Flavonols total (chromatographic method)	21.2 mg/100 g [12], 273.96 mg/100 g DM [54], 34.7 mg/100 g FW frozen [63], 35.0 mg/100 g FW blanched [63], 71 mg QRE/100 g FW [72], 76.43 mg/kg [74], 192.4–408.4 mg/kg FW [75]	16.5–21.3 mg/100 g FW ^X^ [63]	57.0–126.8 mg/100 g DM dried pomace fractions [19], 16.7 mg/100 g FW presscake ^X^ [63], 0.021 g QRE/l juice concentrate [87], 308.9 mg QRE/100 g juice concentrate [107], 4.3–16.4 mg QE/100 mL liqueur [102], 23.93 mg QE/g extract [97]
Kaempferol-3-*O*-galactoside	0.54 mg KE/kg [61]	-	-
Isorhamnetin-3-*O*-galactoside	ND ^1^, 1.5 mg IE/kg [61]	-	-
Isorhamnetin-3-*O*-glucoside	ND ^1^, 1.2 mg IE/kg [61]	-	ND extract [113]
Isorhamnetin-3-*O*-rhamnosylhexoside	-	0.81–4.80 IGaE mg/L [40]	-
Isorhamnetin pentosylhexoside	-	0.30–0.81 mg/100 g DM [33]	4.33 mg/100 g DM fruit powder [33], 0.81–12.20 mg/100 g DM pomace powder [33]
Isorhamnetin pentoside hexoside	1.12 mg/100 g FW [62]	-	-
Isorhamnetin rhamnosylhexosideisomer sum	-	0.74–1.85 mg/100 g DM [33]	18.41 mg/100 g DM fruit powder [33], 7.95–14.27 mg/100 g DM pomace powder [33]
Isorhamnetin 3-*O*-neohesperidoside	1.16 mg/100 g FW [62]	-	-
Isorhamnetin 3-*O*-rutinoside	ND [61], 0.83 mg/100 g FW ^Z^ [62]	-	-
Myricetin-3-*O*-galactoside	0.55 mg ME/kg [61], ND [62]	-	ND extract [113]
Myricetin-3-*O*-glucoside	0.20 mg ME/kg [61], ND [62]	-	ND extract [113]
Myricetin-glucoside/galactoside	0.03 mg ME/100 g FW [51]	-	-
Quercetin-3-*O*-arabinopyranoside Quercetin 3-arabinoside	ND [51], 5.0 mg QGaE/kg [61], 3.1 mg/100 g FW [62]	-	ND extract [70]
Morin (3,5,7,2′,4′-Pentahydroxyflavonol)	-	-	ND-0.501 mg/g tea infusion ^Z^ [108]
Kaempferol	ND [62], 5.30 mg/kg ^Z^ [74]	-	14.5 µg/100 g herb honey ^Z^ [69], 1.12 mg/100 g DM dried fruits ^Z Y^ [99]
Kaempferol-3-*O*-glucoside (Astragalin)	ND [12], ND [61], 0.38 mg/100 g FW ^Z^ [62]	-	ND extract [70], ND extract [113]
Kaempferol-glucoside/galactoside	0.40 mg KE/100 g FW [51]	-	-
Quercetin-3-*O*-galactoside (Hyperoside) 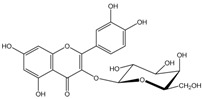	6.6–9.9 mg/100 g [1], 8.31 mg/100 g [12], 9.91–14.57 mg QRE/100 g FW [13], 8.90 mg QE/100 g FW [51], 36.98 mg QRE/100 g DM [53], 65.6 mg/kg ^Z^ [61], 19.09 mg/100 g FW ^Z^ [62], 10.6 mg QRE/100 g FW frozen [63], 10.2 mg QRE/100 g FW blanched [63], 0.320–0.558 mg/g DM ^Z^ [67], 28.3 mg QGlE/100 g FW [110]	6.77–16.46 mg/100 g DM [33], 49.76 mg QRE/100 g DM [53], 4.1–5.7 mg QRE/100 g FW ^X^ [63], 7.0–10.3 mg/L ^Z^ [111], 0.05 mg QGlE/g [78], 97 mg/L ^Z^ [79], 9.8 mg/100 g [82], 76.0–94.8 µg/mL ^Z^ [101], 2.83 mg QRE/100 mL [112]	28.1–62.5 mg QRE/100 g DM dried pomace fractions [19], 104.11 mg/100 g DM fruit powder [33], 48.97–102.43 mg/100 g DM pomace powder [33], 47.44 mg QRE/100 g DM pomace [53], 0.17–0.27 mg/g DM extract ^Z^ [57], 5.2 mg QRE/100 g FW presscake ^Z^ [63], 2.83 g/kg extract [70], 7.68 mg QRE/g DM extract [113], 0.00032 g QRE/l juice concentrate [87], 0.06 mg QGlE/g compote [78], 0.31 mg QGlE/g dried fruit [78], 7.2 mg/g extract [91], 0.43 mg/100 mL fruit tea (decoction) ^Z^ [95], 0.27 mg/100 mL fruit tea (infusion) ^Z^ [95], 4.64 mg/g extract [96], 8.9 mg/g extract [98]
Quercetin-3-*O*-glucoside (Isoquercitrin) 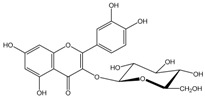	4.4–11.3 mg/100 g [1], 4.03 mg/100 g [12], 7.07–8.87 mg QRE/100 g FW [13], 15.27 mg QE/100 g FW [51], 21.64 mg QRE/100 g DM [53], 43.8 mg QGaE/kg [61], 12.73 mg/100 g FW ^Z^ [62], 7.6 mg QRE/100 g FW frozen [63], 7.2 mg QRE/100 g FW blanched [63], 0.239–0.424 mg/g DM ^Z^ [67], 20.8 mg/100 g FW ^Z^ [110]	7.08–13.54 mg/100 g DM [33], 31.24 mg QRE/100 g DM [53], 3.2–4.2 mg QRE/100 g FW ^X^ [63], 21.4 mg/L ^Z^ [35], 4.8–5.8 mg/L ^Z^ [111], 0.03 mg/g ^Z^ [78], 53 mg/L ^Z^ [79], 4.8 mg/100 g [82], 2.25 mg QRE/100 mL [112]	63.27 mg/100 g DM fruit powder [33], 32.75–67.14 mg/100 g DM pomace powder [33], 26.50 mg QRE/100 g DM pomace [53], 0.10–0.15 mg/g DM extract ^Z^ [57], 3.6 mg QRE/100 g FW presscake ^X^ [63], 2.25 g/kg extract [70], 0.00050 g QRE/l juice concentrate [87], 0.22 mg/g dried fruit ^Z^ [78], 0.03 mg/g compote ^Z^ [78], 5.8 mg/g extract [91], 0.28 mg/100 mL fruit tea (decoction) ^Z^ [95], 0.22 mg/100 mL fruit tea (infusion) ^Z^ [95], 4.02 mg/g extract [96], 21.5 mg/g extract [98]
Quercetin-3-*O*-glucoside + Quercetin-3-*O*-rutinoside	-	-	16.7–37.4 mg QRE/100 g DM dried pomace fractions [19]
Quercetin-dihexoside	33.3 mg QGaE/kg [61], 5.67 mg/100 g FW [62], 4.4 mg QRE/100 g FW frozen [63], 5.2 mg QRE/100 g FW blanched [63]	2.89–6.36 mg/100 g DM [33], 2.3–3.3 mg QRE/100 g FW ^X^ [63], 5.00–29.39 mg QGlE/l [40]	43.58 mg/100 g DM fruit powder [33], 21.99–43.15 mg/100 g DM pomace powder [33], 2.3 mg QRE/100 g FW presscake ^X^ [63]
Quercetin-3-*O*-rutinoside (Rutin) 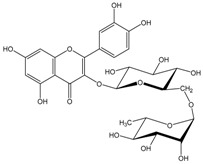	3.9–6.1 mg/100 g [1], 5.51 mg/100 g [12], 5.50–6.27 mg/100 g FW ^Z^ [13], 14.1 mg QE/100 g FW ^Z^ [51], 15.10 mg/100 g DM ^Z^ [53], 42.4 mg QGaE/kg [61], 5.13 mg/100 g FW ^Z^ [62], 3.9 mg QRE/100 g FW frozen ^Z^ [63], 4.1 mg QRE/100 g FW blanched ^Z^ [63], 0.158–0.189 mg/g DM ^Z^ [67], 12.6 mg QGlE/100 g FW [110], 192.1–403.0 mg/kg FW ^Z^ [75]	4.29–8.98 mg/100 g DM [33], 27.53 mg/100 g DM ^Z^ [53], 2.3–2.8 mg/100 g FW ^Z X^ [63], 13.33–53.42 QGlE mg/L [40], 70.9 mg/L ^Z^ [35], 5.9–6.9 mg/L ^Z^ [111], 107.13 mg/L ^Z^ [76,77], 194 mg/L ^Z^ [79], 3.4 mg/100 g [82], 93.6–141.7 µg/mL ^Z^ [101], 1.68 mg/100 mL ^Z^ [112]	44.31 mg/100 g DM fruit powder [33], 22.74–43.68 mg/100 g DM pomace powder [33], 13.55 mg/100 g DM pomace ^Z^ [53], 0.31–0.42 mg/g DM extract ^Z^ [57], 1.7 mg/100 g FW presscake ^Z X^ [63], 1.68 g/kg extract [70], 16.77 mg/g DM extract [113], 0.00091 g/L juice concentrate ^Z^ [87], 0.032–0.738 mg/g tea infusion ^Z^ [108], 153.8 mg/kg extract ^Z^ [114], 79.95–100.21 mg/L juice concentrate ^Z^ [76], 86.37–97.69 mg/L juice concentrate ^Z^ [77], 0.08–0.10 mg/g DM dried fruits [90], 5.2 mg/g extract [91], 0.94 mg/100 mL fruit tea (decoction) ^Z^ [95], 0.58 mg/100 mL fruit tea (infusion) ^Z^ [95], 498.80 mg/L extract ^Z^ [109], 18.3 mg/g extract [98]
Quercetin-3-*O*-glucuronide (Miquelianin)	5.6 mg QGaE/kg [61], ND [62]	-	ND extract [113]
Quercetin-3-*O*-vicianoside (Peltatoside) 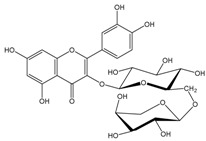	2.6–4.3 mg/100 g [1], 2.36 mg/100 g [12], 3.84–5.41 mg QRE/100 g FW [13], 36.4 mg QGaE/kg [61], 4.0 mg QRE/100 g FW frozen [63], 4.5 mg QRE/100 g FW blanched [63], 8.5 mg QGlE/100 g FW [110]	1.95–5.50 mg/100 g DM [33], 2.5–3.1 mg QRE/100 g FW ^X^ [63], 8.80–43.48 QGlE mg/L [40], 1.8–2.0 mg/L [111], 1.15 mg QRE/100 mL [112]	45.32 mg/100 g DM fruit powder [33], 20.41–43.20 mg/100 g DM pomace powder [33], 1.9 mg QRE/100 g FW presscake ^X^ [63], 1.15 g/kg extract [70], 3.78 mg QRE/g DM extract [113]
Quercetin-3-*O*-xyloside	0.40 mg QE/100 g FW [51], 1.5 mg QGaE/kg [61], ND [62]	-	ND extract [70]
Quercetin-3-*O*-robinobioside 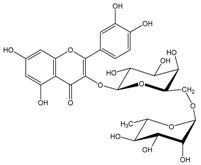	1.1–11.3 mg/100 g [1], 1.03 mg/100 g [12], 5.42–5.76 mg QRE/100 g FW [13], 29.6 mg QGaE/kg [61], 3.5 mg QRE/100 g FW frozen [63], 3.1 mg QRE/100 g FW blanched [63], 11.1 mg QGlE/100 g FW [110]	4.94–10.75 mg/100 g DM [33], 1.6–2.1 mg QRE/100 g FW ^X^ [63], 23.59–118.89 QGlE mg/L [40], 1.17 mg QRE/100 mL [112]	47.45 mg/100 g DM fruit powder [33], 25.60–50.52 mg/100 g DM pomace powder [33], 1.4 mg QRE/100 g FW presscake ^X^ [63], 1.17 g/kg extract [70]
Quercetin-*O*-dihexoside	-	0.19–0.58 mg/100 g DM [33]	1.96 mg/100 g DM fruit powder [33], 1.31–3.04 mg/100 g DM pomace powder [33]
Quercetin-*O*-deoxyhexoside-hexoside	3.64 mg/100 g FW [62]	-	-
Quercetin hexoside pentoside	-	-	0.24 g QRE/l juice concentrate [87]
Quercetin-arabinoglucoside Quercetin 3-*O*-arabinoglucoside	6.63 mg/100 g FW [62]	0.22 mg QGlE/g [78]	0.13 mg QGlE/g dried fruit [78], 0.02 mg QGlE/g compote [78]
Quercetin 3-*O-*(6”-malonyl)-glucoside	1.52 mg/100 g FW ^Z^ [62]	-	ND extract [113]
Quercetin	0.21 mg/100 g FW ^Z^ [51], ND [62], 0.74 mg/100 g FW frozen ^Z^ [63], 0.66 mg/100 g FW blanched ^Z^ [63], 71.13 mg/kg ^Z^ [74], 0.3–17.4 mg QRE/kg FW [75]	0.19–0.40 mg/100 g FW ^Z X^ [63], 1.75–22.73 mg/L [40], 64.4 mg/L ^Z^ [35], 0.27 mg QRE/l [76,77], 11.8 mg/100 mL [83], ND-26.9 µg/mL ^Z^ [101]	ND [8,9], 6.7–16.4 mg/100 g DM dried pomace fractions ^Z^ [19], 0.66 mg/100 g FW presscake ^Z X^ [63], 11.6 µg/100 g herbhoney ^Z^ [69], 0.00006 g QRE/l juice concentrate [87], ND-0.243 mg/g tea infusion ^Z^ [108], 1.9 mg/kg extract ^Z^ [114], 1.6 mg/g extract [91], 117.60 mg/L extract ^Z^ [109], 1.56 mg/g extract [96], 1.8 mg/g extract [98], 42.28 mg/100 g DM dried fruits ^Z Y^ [99]
Quercetin-diglucoside	9.24 mg QE/100 g FW [51]	-	-
Chrysin (5,7-Dihydroxyflavone)	-	-	3.5 µg/100 g herbhoney ^Z^ [69]
Hesperetin	-	-	10.5 µg/100 g herbhoney ^Z^ [69]
Naringenin	-	-	31.3 µg/100 g herbhoney ^Z^ [69]
Flavan-3-ols total/sum	-	-	7281–13504 mg/100 g DM dried pomace fractions [19], 6.6 g CE/l juice concentrate [87], 3940.1 µg CE/g extract [89], 93.90 mg/g extract [97]
(-)-Epicatechin 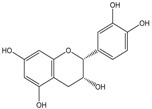	467.35–862.50 mg/kg FW [3], 15.76–32.18 mg/100 g FW [13], 0.12 mg/100 g FW ^Z^ [51], 15.04 mg/100 g DM [53], ND [62]	12.71 mg/100 g DM [53], 213.58–235.28 mg/100 g DM ^Z^ [33], 40.2 mg CE/l [35], 1.48 mg/100 mL [112]	6.6–12.0 mg/100 g DM dried pomace fractions [19], 174.53 mg/100 g DM fruit powder ^Z^ [33], 236.19–260.13 mg/100 g DM pomace powder ^Z^ [33], 11.41 mg/100 g DM pomace [53], 1.95 g/kg extract [70], ND extract [113], 12.77 mg/g extract ^Z^ [97], 7.6 mg/g extract [98]
(+)-Catechin	ND [51,62]	87.66–107.18 mg/100 g DM ^Z^ [33]	122.70 mg/100 g DM fruit powder ^Z^ [33], 142.81–180.27 mg/100 g DM pomace powder ^Z^ [33], ND extract [70], ND extract [113], 19.93 mg/g extract ^Z^ [97]
Proanthocyanidins total (spectrophotometric method)	8–178 g CE/kg FW [11], 2.46–3.74 g PCB2E/100 g FW [56], 845.2 mg CE/100 g FW frozen [63], 868.6 mg CE/100 g FW blanched [63], 9.25–13.5 mg CE/g DM [67]	4.6–15 g CE/kg FW [11], 392.6–464.8 mg CE/100 g FW ^X^ [63], 60–72 CyE mg/100 mL [45], 3529.1 mg CE/l [47], 0.64–4.17 g CyE/l [40], 240 mg CE/l [79], 34.2 mg CE/100 mL [81], 442 mg CE/100 g [82], 3122.5 mg/L [85]	524.2 mg CE/100 g FW presscake ^X^ [63], 30.87–59.22 mg CE/100 mg DM powder [88], 5.6 mg CE/g extract [89], 83.8 mg CE/g extract [91], 129.87 mg/g extract [92], 305 mg CE/g DM extract [93], 39.2 mg CE/g extract [93], 24–129 g CE/kg FW pomace [93]
Proanthocyanidins total/sum (chromatographic method)	1426.66–1645.64 mg/100 g FW [13], 80.50 mg ECE/100 g FW [51], 5181.60 mg/100 g DM [53], 663.7 mg/100 g FW [59], 1564–3259μg CE/g DM [67]	1472.27–2371.07 mg/100 g DM [33], 1578.79 mg/100 g DM [53], 3926.2 mg/L [84], 293.38 mg/100 mL [112]	7274–13492 mg/100 g DM dried pomace fractions [19], 9977.84 mg/100 g DM fruit powder [33], 6201.73–9714.57 mg/100 g DM pomace powder [33], 8191.58 mg/100 g DM pomace [53], 390.0 mg CGlE/kg extract [114], 532.8 mg/100 g juice concentrate [107], 5.14 mg CE/g extract [91], 146.4 mg/g extract [98]
Mean degree of polymerisation (mDP) polymeric procyanidins	19 [11], 42–59 [13], 23 [53]	24 [11], 23 [53], 41 [45], 12–52 [40]	18 pomace [11], 15.5–37.5 dried pomace fractions [19], 34 pomace [53]
Mono-, di-, oligomeric flavan-3-ols	326.55 mg/100 g DM [54]	-	-
Polymeric proanthocyanidins	3816.36 mg/100 g DM [54], 1562–3258 µg CE/g DM [67]	-	14.9 g/kg extract [70]
Procyanidin B1	-	-	10.27 mg/g extract ^Z^ [97]
Procyanidin B2	-	21.90–28.19 mg/100 g DM ^Z^ [33]	24.86 mg/100 g DM fruit powder ^Z^ [33], 36.40–42.13 mg/100 g DM pomace powder ^Z^ [33], ND extract [70], ND extract [97]
Monomers	5.89 mg ECE/100 g FW [51], 5.17 mg/100 g FW [59], 0.01–0.02 µg CE/g DM [67]	-	60.0 mg/100 g juice concentrate [107], 0.0088 mg CE/g extract [91]
Dimers	0.57 mg ECE/100 g FW [51], 12.48 mg/100 g FW [59], 0.10–0.39 µg CE/g DM [67]	ND-423 µg/mL [101]	101.5 mg/100 g juice concentrate [107], 0.36 mg CE/g extract [91]
Trimers	0.79 mg ECE/100 g FW [51], 10.29 mg/100 g FW [59], 0.22–0.86 µg CE/g DM [67]	-	69.6 mg/100 g juice concentrate [107], 1.66 mg CE/g extract [91]
4˗6-mers	40.32 mg/100 g FW [59], 0.48–1.75 µg CE/g DM [67]	-	-
Tetramers	0.70 mg ECE/100 g FW [51]	-	83.4 mg/100 g juice concentrate [107], 1.24 mg CE/g extract [91]
Pentamers	0.75 mg ECE/100 g FW [51]	-	68.8 mg/100 g juice concentrate [107], 0.76 mg CE/g extract [91]
Hexamers	1.04 mg ECE/100 g FW [51]	-	129.9 mg/100 g juice concentrate [107], 0.34 mg CE/g extract [91]
7˗10-mers	52.87 mg/100 g FW [59], 0.10–0.36 µg CE/g DM [67]	-	-
Heptamers	0.56 mg ECE/100 g FW [51]	-	60.7 mg/100 g juice concentrate [107], 0.16 mg CE/g extract [91]
Oktamers	0.51 mg ECE/100 g FW [51]	-	0.064 mg CE/g extract [91]
≥Nonamers	-	-	0.54 mg CE/g extract [91]
Decamers	0.16 mg ECE/100 g FW [51]	-	-
>10-mers	69.0 mg ECE/100 g FW [51], 542.6 mg/100 g FW [59], 1562–3258 µg CE/g DM [67]	-	-

DM: dry matter, FW: fresh weight, ND: not detected, Tr: trace, ^Z^: value calculated from the curve for the standard corresponding to the determined compound, ^X^: concentration on original berry weight basis, ^Y^: after hydrolysis. Equivalents: CaE: caffeic acid, CArE: cyanidin 3-*O*-arabinoside, CE: catechin, CGaE: cyanidin 3-*O*-galactoside, CGlE: cyanidin 3-*O*-glucoside, ChAE: chlorogenic acid, CoAE: *p*-coumaric acid, CRE: cyanidin 3-*O*-rutinoside, CyE: cyanidine, ECE: epicatechin, GAE: gallic acid, IE: isorhamnetin, IGaE: isorhamnetin 3-*O*-galactoside, KE: kaempferol, ME: myricetin, NChAE: neochlorogenic acid, NE: naringenin, PCAE: protocatechuic acid, PCB2E: procyanidin B2, PE: pelargonidin, QE: quercetin, QGaE: quercetin 3-*O*-galactoside, QGlE: quercetin 3-*O*-glucoside, QRE: quercetin 3-*O*-rutinoside, SAE: sinapic acid.

**Table 3:** Antioxidant activity of chokeberry fruits and products.

Product	Experimental model	Method	Result	Reference
Chokeberry juices from different growing seasons	Chokeberries (200 g) were mixed in a house blender. Juice was separated from the mash by subsequent pressing, bottled and stored at 4 °C.	DPPH [mmol TE/l]	juice_2012_ 14.6, juice_2013_ 13.4, juice_2014_ 12.9	Tolić et al. [5]
		FRAP [mmol Fe^2+^/l]	juice_2013_ 179.5, juice_2012_ 166.7, juice_2014_ 128.2	
Chokeberry products	Powdered fruits and pomace (2 g) were extracted with 50 mL of MeOH acidified with 2.0% formic acid. The extraction was performed twice by incubation for 20 min under sonication. Next, the slurry was centrifuged and the supernatant was filtered.	ABTS [mmol TE/100 g DM]	powder from dried fruit 81.66, powder from pomace of uncrushed fruit 81.63, powder from pomace of crushed fruit 59.94, juice from crushed fruit 32.73, juice from uncrushed fruit 20.11	Oszmiański and Lachowicz [33]
		DPPH [mmol TE/100 g DM]	powder from dried fruit 53.78, powder from pomace of uncrushed fruit 52.22, powder from pomace of crushed fruit 32.61, juice from crushed fruit 20.20, juice from uncrushed fruit 9.81	
Dried chokeberry fruits by freeze drying (FD), vacuum-microwave drying (VMD), vacuum drying (VD), convection drying (CD), convection-vacuum-microwave drying (CVM)	Chokeberry powder (5 g) was weighed into a test tube, 25 mL of 80% MeOH with 1% HCl was added, and the suspension was stirred slightly. Tubes were sonicated for 15 min twice and left at 4 °C for 24 h. Afterward, the extract was centrifuged and supernatants were collected.	ABTS [mmol TE/100 g DM]	fresh 234.9, FD 114.7, CVM_2h+360/240_ 83.0, CVM_6h+360/120_ 82.7, CVM_6h+360/240_ 80.5, VMD_120W_ 78.4, CVM_2h+360/120_ 77.9, VMD_240W_ 77.2, VMD_480/120W_ 76.3, VMD_480/240W_ 76.2, VMD_360/240W_ 75.9, VD 75.3, CD_70 °C_ 75.0, VMD_240/120W_ 73.2, VMD_360/120W_ 72.3, CD_60 °C_ 52.4, CD_50 °C_ 41.9	Samoticha et al. [58]
		FRAP [mmol TE/100 g DM]	Fresh 39.0, FD 26.3, CVM_2h+360/240_ 23.8, CVM_6h+360/240_ 22.1, CVM_6h+360/120_ 21.5, VMD_360/240W_ 20.7, VMD_480/240W_ 19.7, VD 19.5, CVM_2h+360/120_ 19.3, VMD_480/120W_ 19.3, VMD_240W_ 19.1, VMD_120W_ 18.5, VMD_240/120W_ 17.8g, CD_70 °C_ 17.7, VMD_360/120W_ 17.5, CD_60 °C_ 16.1, CD_50 °C_ 15.4	
Commercial chokeberry juices	Not specified.	ABTS [mmol TE/1]	20.39–91.21	Sosnowska et al. [40]
		DPPH [mmol TE/1]	19.02–106.13	
		FRAP [mmol TE/1]	12.19–61.09	
Fruit extracts	Freeze-dried fruits were disintegrated with mill and were triplicate extracted with 70% EtOH. Samples were dried, then dissolved in distilled water and percolated through an Amberlite XAD4 column to adsorb polyphenols. Polyphenols were obtained after washing the column with 70% ethanol. The collected fraction was evaporated in a vacuum evaporator until dry mass.	phosphatidylcholine (PC) liposome oxidation inhibition [IC_50_ μg/mL]	chokeberry 24.6, blackcurrant 30.9, rosehip 33.5, hawthorn 45.9	Strugała et al. [113]
Dried pomaces	Dried, milled and sieved fruits pomace (6–10 g) were extracted in two steps with 100 mL of eluent (50 + 50) deionized H_2_O (temp. 100 °C) and left for 5 min. Mixture was shaken for 15 min and then filtered through a 45 μm filter. The extracts were collected and evaporated on a rotary evaporator at 40 °C.	DPPH [%]	blackcurrant 68.2, chokeberry 67.0, apple 47.3, strawberry 39.3, carrot 37.7	Pieszka et al. [16]
		TRAP [μg TE/g]	chokeberry 179, apple 96, black currant 82, strawberry 61, carrot 50	
Selected edible fruits and their leaves	Fruits (0.5 g) and leaves (0.2 g) were freeze dried and extracted by 10 mL of mixture containing MeOH (30 mL/100 mL), ascorbic acid (2.0 g/100 mL) and acetic acid (1.0 mL/100 mL) of reagent. The extracts were sonicated for 15 min, left for 24 h at 4 °C in darkness, sonicated again for 15 min and centrifuged.	ABTS [mM TE/100 g DM]	quince_leaves_ 116.49, cranberry_leaves_ 96.02, bilberry_leaves_ 79.30, Japanese quince_leaves_ 60.30, chokeberry_fruits_ 52.31, chokeberry_leaves_ 50.01, apple_leaves_ 35.94, bilberry_fruits_ 35.34, blackcurrant_leaves_ 32.91, Japanese quince_fruits_ 32.88, blackcurrant_fruits_ 22.47, cranberry_fruits_ 14.61, apple_fruits_ 8.72, quince_fruits_7.85	Teleszko and Wojdyło [54]
		FRAP [mM TE/100 g DM]	quince_leaves_ 65.25, bilberry_leaves_ 59.58, cranberry_leaves_ 43.17, chokeberry_leaves_ 40.55, Japanese quince_leaves_ 40.09, chokeberry_fruits_ 36.64, bilberry_fruits_ 26.81, Japanese quince_fruits_ 19.51, blackcurrant_leaves_ 19.16, apple_leaves_ 15.30, blackcurrant_fruits_ 11.82, cranberry_fruits_ 8.40, quince_fruits_ 5.43, apple_fruits_ 3.44	
Chokeberry products: (juices (J), powders (P), capsules (C), fruit teas (FT), dried berries (DB))	Samples (6 g) were mixed with 20 mL of MeOH/2% HCl (95:5 *v*/*v*). After 60 min the solution was filtered under vacuum in a 50 mL. Extraction of the residue was repeated using the same conditions. The filtrates were combined and adjusted to 50 mL with MeOH/2% HCl (95:5 *v*/*v*).	DPPH [mmol TE/l juice or mmol TE/100 g DM powder, capsules, fruit teas, dried berries]	DB_2_ 191.31, DB_1_ 183.52, FT_3_ 163.33, FT_4_ 153.96, FT_1_ 149.44, P_3_ 131.06, FT_2_ 111.43, P_2_ 105.68, P_1_ 95.00, C_2_ 80.93, C_1_ 58.49, J_10_ 40.19, J_8_ 34.22, J_1_ 33.37, J1_1_ 28.12, J_9_ 26.25, J_2_ 23.03, J_6_ 20.66, J_4_ 19.47, J_5_ 18.29, J_7_ 16.51, J_2_ 12.09	Tolić et al. [34]
		FRAP [mmol of Fe^2+^/l juice or mmol of Fe^2+^/100 g DM powder, capsules, fruit teas, dried berries]	J_6_ 79.86, J_1_ 76.14, J_4_ 72.43, J_8_ 71.50, P_2_ 68.60, C_1_ 65.82, J_10_ 62.92, P_1_ 60.66, C_2_ 60.35, J_11_ 60.13, J_2_ 51.50, J_2_ 48.76, J_5_ 48.64, P_3_ 47.38, FT_2_ 43.12, J_7_ 38.98, J_9_ 38.71, FT_1_ 32.74, DB_1_ 21.51, DB_2_ 17.40, FT_4_ 15.94, FT_3_ 13.50	
Different fruits extracts	Powdered, freeze dried fruits (5 g) were extracted with 100 mL 80% acetone in 0.2% formic acid at room temperature for 1 h. After that, the samples were centrifuged and concentrated via rotary evaporation to a volume of 15 mL in order to fully remove the acetone. Then the volume was adjusted to 50 mL with ultra clean H_2_O and the extracts were centrifuged.	ORAC [μmol TE/l]	rosehip 93677.6; hawthorn 73804.6; blueberry 72487.2; chokeberry 55505.7; blackcurrant 46421.7; rowanberry 23689.6	Denev et al. [122]
		TRAP [μmol TE/l]	rosehip 87109.4; hawthorn 51125.1; blueberry 43433.6; chokeberry 43217.1; rowanberry 34612.0; blackcurrant 33510.6	
		H-ORAC [μmol GAE/L]	rosehip 76069.4; hawthorn 31328.5; blueberry 26339.0; chokeberry 22506.0; blackcurrant 20019.3; rowanberry 15373.4	
		Lipid peroxidation [% of control]	blackcurrant> rowanberry> blueberry > chokeberry > rosehip > hawthorn autoxidation of linoleic acid was effectively inhibited by blueberry, rowanberry and blackcurrant extracts at the end of the first day of storage. The inhibition of hydroperoxide formation by these fruits and, in addition by chokeberry, was observed also after the third day of linoleic acid autoxidation (less than 50% of control). Finally, hydroperoxide formation was inhibited to more than 50% of the control value by all extracts analysed after 6 days of linoleic acid autoxidation. Extract from blueberry was the most effective as it diminished the hydroperoxides to 8% of control value.	
Dried (D) and candied (C) fruits	Dried and homogenized whole edible parts of fruits (15 g) were dispersed in 20 mL of 62.5% aqueous methanol containing 2 g/L of TBHQ. To this extract 5 mL of 6M HCl was added, the hydrolysis was carried out in a shaking water bath at 85 °C for 2 h. Then the sample was filtered, made up to 50 mL with methanol, and sonicated (5 min.).	ABTS [mmol/100 g DM]	chokeberries_D_ 21.378, bilberries_D_ 17.996, cherries_C_ 3.038, plums_D_ 2.913, grapes (amber light) _D_ 2.188, apricots_D_ 1.377, cranberries_C_ 0.835, grapes (amber dark)_D_ 0.648, dates_C_ 0.621f, figs_D_ 0.388	Miletić et al. [99]
		DPPH [μmol/100 g DM]	bilberries_D_ 2130.23, chokeberries_D_ 1815.08, plums_D_ 503.65, dates_C_ 388.98, apricots_D_ 317.56, grapes (amber light) _D_ 264.56, cherries_C_ 254.64, grapes (amber dark) _D_ 152.53, cranberries_C_ 139.80, figs_D_ 129.55	
Wild and cultivated small fruits	Frozen fruits (6 g) were homogenized and mixed with 10 mL of ethyl acetate. The procedure was repeated four times. Extract (20 mL) was evaporated to dryness and the residue was dissolved in 4 mL of MeOH.	DPPH [μmol TE/mg DM]	blackberry 0.2125, chokeberry 0.1065, cherry 0.1030, blackthorn 0.0785, raspberry 0.0725	Mitic et al. [123]
		ABTS [μmol TE/mg DM]	blackberry 0.3616, cherry 0.2552, chokeberry 0.1808, blackthorn 0.1704, raspberry 0.1576	
		total reducing power [μmol AAE/mg DM]	blackberry 0.1920, cherry 0.1720, aronia 0.1540, blackthorn 0.1440, raspberry 0.1180	
		FRAP [μmol Fe/mg DM]	blackberry 1.0900, aronia 0.6120, cherry 0.5660, blackthorn 0.4100, raspberry 0.3780	
Different fruits	Fruits (20 g) were extracted with 70% acetone (200 mL) at room temperature for 60 min with stirring. After centrifugation, and filtration, the supernatants were concentrated by vacuum rotary evaporator. The aqueous phase was diluted to 25 mL with H_2_O.	ABTS [μmol TE/g]	chokeberry 124.66, bilberry 54.17, blue honeysuckle 51.54, blackcurrant 35.75, lingonberry 34.82, blackberry 28.91, blueberry 27.09, red gooseberry 24.39, red currant 23.45, cranberry 20.43, raspberry 20.36, green gooseberry 18.56, strawberry 16.56, pomegranate 14.02, sour cherry 10.70, grape pink 10.38, apple 8.77, sweet cherry 6.00, orange 4.80, pineapple 4.02, red grapefruit 3.69, mandarine 3.54, pomelo 3.25, plum ‘Węgierka Zwykła’ 2.03, kiwi 1.97, banana 1.83, pear ‘Nashi’ 1.65, peach 1.59, pear ‘Lukasówka’ 1.46, plum ‘Renkloda’ 0.84	Podsędek et al. [124]
		FRAP [μmol TE/g]	chokeberry 94.24, blue honeysuckle 49.52, bilberry 41.70, blackcurrant 29.93, blackberry 23.36, lingonberry 22.28, red gooseberry 16.99, blueberry 16.86, raspberry 16.81, red currant 16.68, cranberry 12.74, green gooseberry 12.28, strawberry 9.95, sour cherry 9.12, pomegranate 7.62, grape pink 6.10, apple 4.98, sweet cherry 4.34, pineapple 4.01, orange 3.50, red grapefruit 3.08, pomelo 2.22, mandarine 1.66, plum ‘Węgierka Zwykła’ 1.49, kiwi 1.46, banana 1.15, pear ‘Lukasówka’ 0.81, peach 0.79, pear ‘Nashi’ 0.76, plum ‘Renkloda’ 0.66	
Commercially available *Aronia melanocarpa* tea infusions (TI)	Tea sample (2 g) were infused with 200 mL deionized H_2_O heated to 95 °C for 10 min. The solutions were filtered and washed with deionized H_2_O, cooled to room temperature and diluted to 250 mL with deionized H_2_O.	DPPH [mmol TE/g]	TI_2_ 0.074, TI_3_ 0.068, TI_1_ 0.067, TI_5_ 0.058, TI_4_ 0.055	Veljković et al. [108]
		ABTS [mmol TE/g]	TI_2_ 2.744, TI_3_ 2.731, TI_1_ 2.715, TI_5_ 0.089, TI_4_ 0.076	
		FRAP [mmol Fe/g]	TI_4_ 0.153, TI_1_ 0.147, TI_3_ 0.147, TI_5_ 0.144, TI_2_ 0.136	
		reducing power [mmol AAE/g]	TI_4_ 3.48, TI_5_ 2.14, TI_1_ 1.36, TI_3_ 0.88, TI_2_ 0.53	
Different fruits	Homogenized fruits (2 g) were mixed with 20 mL of 0.2% formic acid in 80% acetone solution. Extraction was conducted at room temperature for 1 h. After that, the samples were centrifuged and supernatants were removed. The solid residues were subjected to the second extraction under the same conditions. Both supernatants were combined.	ORAC [μmol TE/g FW]	elderberry 205.4, brier 201.1, chokeberry 160.8, hawthorn 153.6, blueberry 98.8, blackcurrant 96.0, rowanberry 80.9, blackthorn 79.1, blackberry 74.2, cranberry 70.0, sour cherry 58.6, cornel cherry 49.0, strawberry 47.2, raspberry 38.9, red grapes 26.8, cherry 25.8, pomegranate 19.7, apple 13.8, fig 13.6, plum 10.8, apricot 7.2, white grapes 6.3, peach 6.2, pumpkin 4.9, watermelon 3.8, honeydew melon 2.3	Denev et al. [50]
Black chokeberry (*Aronia melanocarpa*) powders from commercial pure clack chokeberry juice (Rabenhorst, Germany), obtained by different drying processes: freeze drying (FD), spray drying (SD), oven vacuum drying (OV)	Juice (5 l) was centrifuged (15 min, 5950× g). The supernatant was loaded into XAD-16 column, sugars were removed by H_2_O, phenolic compounds were removed by EtOH. The remaining solvent was evaporated at 40 °C up to the final volume of aqueous extract 2 l. Extracts was subjected to different drying processes. Dry powders was resolubilized with 5 mL of 20% MeOH (*v*/*v*) by 60 sec sonication followed 60 sec of vortexing. The procedure was repeated (3 ×). In case of PCL assay extraction of the hydrophilic antioxidants required about 10 mg of the powders followed by the addition of 10 mL of deionized H_2_O treated in an ultrasonic bath for 2 min, vortexed for 1 min (3 ×) and centrifuged. The same procedure was applied to extraction of lipophilic compounds with MeOH. All extracts obtained were filtered (0.45μm PTFE filter).	ABTS [μmol TE/100 mg DM]	SD 251.34, FD 180.45, OV_40 °C_ 175.85, OV_60 °C_ 165.47, OV_80 °C_ 158.08	Horszwald et al. [88]
		DPPH [μmol TE/100 mg DM]	SD 26.49, FD 24.68, OV_40 °C_ 22.81, OV_60 °C_ 20.20, OV_80 °C_ 15.80	
		FRAP [μmol TE/100 mg DM]	SD 248.56, FD 193.69, OV_60 °C_ 179.91, OV_80 °C_ 171.38, OV_40 °C_ 165.27	
		PCL ACW [μmol TE/100 mg DM]	OV_40 °C_ 291.94, OV_60 °C_ 289.79, FD 282.34, SD 279.33, OV_80 °C_ 238.72	
		PCL ACL [μmol TE/100 mg DM]	FD 476.15, OV_40 °C_ 447.06, OV_60 °C_ 436.79, OV_80 °C_ 427.36, SD 411.73	
Black chokeberry fruits and products	Sample (2 g) was extracted in a cooled ultrasonic bath for 15 min using 5 mL of 75% MeOH containing 0.1% (*v*/*v*) formic acid. Samples were then centrifuged for 10 min at 83 Hz and the supernatant was collected. This procedure was repeated four times until the total volume reached 20 mL.	ABTS [g TE/kg FW]	dried fruit_1_ 74.0, dried fruit_2_ 54.4, pomace 49.6 concentrate 22.0, fruit 11.0, juice_2_ 10.8, juice_3_ 10.8, juice_1_ 9.8, jam_2_ 9.8, compote 9.4, jam_1_ 9.0, syrup 3.7, sour cherry-chokeberry syrup 2.0, raspberry-chokeberry syrup 1.2	Kapci et al. [7]
		DPPH [g TE/kg FW]	dried fruit_1_ 36.3, dried fruit_2_ 30.5, pomace 25.2, fruit 11.3, concentrate 10.8, jam_2_ 8.7, juice_2_ 6.2, juice_3_ 5.8, juice_1_ 5.7, jam_1_ 5.0, compote 4.8, syrup 2.2, sour cherry-chokeberry syrup 2.0, raspberry-chokeberry syrup 0.7	
		CUPRAC [g TE/kg FW]	dried fruit_1_ 257.2, dried fruit_2_ 233.2, pomace 192.4, concentrate 74.5, fruit 67.7, jam_2_ 57.4, juice_2_ 35.1, juice_1_ 33.8, jam_1_ 33.6, compote 33.2, juice_3_ 30.7, syrup 13.4, sour cherry-chokeberry syrup 5.2, raspberry-chokeberry syrup 3.0	
Berry fruit ethanol extracts	Not specified.	ABTS [µM TE/g FW]	blackcurrant ‘Titania’ 56.8, chokeberry ‘Nero’ 53.2, blackberry ‘Polar’ 51.7, raspberry ‘Polana’ 28.5, highbush blueberry ‘Bluecrop’ 27.3, red chokeberry ‘Brilliant’ 23.8, red currant ‘Heros’ 22.1, white currants ‘Blanca’ 7.4	Najda and Łabuda [73]
		DPPH [µM TE/g FW]	chokeberry ‘Nero’ 199.4, blackcurrant ‘Titania’138.90, blackberry ‘Polar’ 129.3, red chokeberry ‘Brilliant’ 63.2, raspberry ‘Polana’ 59.4, red currant ‘Heros’ 48.3, highbush blueberry ‘Bluecrop’ 40.4, white currants ‘Blanca’ 19.4	
		FRAP [µM Fe/g FW]	chokeberry ‘Nero’ 112.5, blackcurrant ‘Titania’ 108.4, blackberry ‘Polar’ 97.1, highbush blueberry ‘Bluecrop’ 34.5, red chokeberry ‘Brilliant’ 34.1, red currant ‘Heros’ 31.7, raspberry ‘Polana’ 27.5, white currants ‘Blanca’ 12.3	
Berry extracts: aqueous (W), 100% acetone (Ac) and 100% hexane (He)	Lyophilized berries were extracted with water, 100% of acetone and 100% of hexane (concentration 25 mg/mL) at room temperature twice during 3 h.	ABTS [μM TE/g]	murtilla non-ripe 620.7, murtilla ripe 446.6, blueberries Poland 254.8, chokeberry 219.3, murteola ripe 200.6, blueberries Chile 197.7, murteola non-ripe 144.4, raspberries 82.5, murtilla non-ripe 56.42, murtilla ripe 29.89, blueberries Poland 26.59, murteola non-ripe 19.79, chokeberry 15.14, murteola ripe 10.08, blueberries Chile 9.79, raspberries 7.61, raspberries 3.25, chokeberry 2.80, murtilla non-ripe 2.30, murteola non-ripe 1.54, blueberries Chile 1.11, murtilla ripe 1.10, blueberries Poland 0.98, murteola ripe 0.60	Arancibia-Avila et al. [125]
		DPPH [μM TE/g]	murtilla non-ripe 334.7, murtilla ripe 208.9, murteola ripe 102.4, blueberries Chile 94.5, chokeberry 87.2, blueberries Poland 75.1, murteola non-ripe 64.6, raspberries 27.7, murtilla non-ripe 23.76, murtilla ripe 16.43, blueberries Poland 15.01, murteola non-ripe 9.32, murteola ripe 4.00, chokeberry 3.55, raspberries 3.29, blueberries Chile 3.01, raspberries 2.14, chokeberry 1.19, murtilla non-ripe 1.15, murteola non-ripe 0.98, blueberries Poland 0.44, blueberries Chile 0.37, murtilla ripe 0.35, murteola ripe 0.18	
		FRAP [μM TE/g]	murtilla non-ripe 327.3, murtilla ripe 208.9, blueberries Poland 177.3, murteola ripe 76.0, blueberries Chile 73.3, chokeberry 57.4, murteola non-ripe 43.0, murtilla non-ripe 41.46, raspberries 27.7, murtilla ripe 20.87, blueberries Poland 11.59, murteola non-ripe 8.38, blueberries Chile 5.59, murteola ripe 2.94, chokeberry 2.84, raspberries 2.80, raspberries 1.99, chokeberry 0.89, murtilla non-ripe 0.89, blueberries Chile 0.58, murteola non-ripe 0.52, blueberries Poland 0.31, murtilla ripe 0.30, murteola ripe 0.16	
		CUPRAC [μM TE/g]	murtilla non-ripe 600.5, murtilla ripe 428.5, blueberries Poland 250.9, chokeberry 212.9, blueberries Chile 154.0, murteola ripe 116.8, murteola non-ripe 82.9, murtilla non-ripe 38.79, blueberries Poland 33.27, raspberries 30.4, murtilla ripe 28.65, murteola non-ripe 14.11, blueberries Chile 13.45, murteola ripe 7.72, chokeberry 7.16, raspberries 6.09, raspberries 4.56, murtilla non-ripe 3.18, chokeberry 3.12, murteola non-ripe 2.86, blueberries Poland 2.78, blueberries Chile 2.68, murtilla ripe 0.77, murteola ripe 0.12	
Different fruits	Fruits were extracted with H_2_O containing 200 ppm of SO_2_ (ratio of solvent to fruits 3:10). Then the extract was adsorbed on Purolite AP 400 resin for further purification. The polyphenols were then eluted out with 80% EtOH, concentrated and freeze dried.	ABTS [µmol TE/mg DM]	apple 5.65, strawberry 4.80, chokeberry 4.15	Bonarska-Kujawa et al. [70]
		Linoleic acid oxidation [antioxidant activity index min/(µg DM/mL)]	apple 47.1, chokeberry 44.4, strawberry 43.1	
		Erythrocyte membranes UV oxidation [I_50_ mg/mL DM]	Trolox 0.0146, apple 0.0286, chokeberry 0.0520, strawberry 0.0529	
		Erythrocyte membranes AAPH oxidation [I_50_ mg/mL DM]	Trolox 0.00390, apple 0.00794, chokeberry 0.00955, strawberry 0.02423	
Wild chokeberry and cultivars	Homogenized berries (15 g) were extracted (3 × 15mL) in the EtOH solution containing 1% hydrochloric acid. Combined extracts were shaken for 30 min and left to stand 24 h. Than the extracts were evaporated using a rotary evaporator.	DPPH [EC_50_ g fruit/g DPPH]	wild 0.46, ‘Nero’ 0.91, ‘Viking’ 0.94, ‘Galicianka’ 2.46	Jakobek et al. [2]
Fruit juices	Fresh juice (1 mL) was diluted up to the volume of 25 mL. Part of solution was centrifuged at 15,000 rpm for 20 min at 4 °C. Supernatant solution was used for analysis. Additionally, non-centrifuged juices were analysed.	ORAC [μmol TE/100 mL]	non-centrifuged samples: black chokeberry 1086.60, blackcurrant 500.80, red currant 422.60, apple 389.40, cranberry 379.70, pomegranate 340.50, blueberry 297.80, lime 285.10, lemon 223.10, grapefruit 105.04, aloe vera 81.35, red orange 71.72, black grapes 58.60, kumquat 38.16, white grapes 30.82 Centrifuged samples: blackcurrant 1271.80, black chokeberry 666.60, red currant 540.50, cranberry 336.60, blueberry 206.80, grapefruit 200.30, apple 196.40, pomegranate 151.40, kumquat 133.50, lemon 125.90, lime 111.90, black grapes 94.30, red orange 35.68, white grapes 31.10, aloe vera 27.07	Keskin-Šašić et al. [126]
Different berry extracts	Lyophilized berries were extracted with MeOH (concentration 25 mg/mL) at room temperature twice during 3 h.	ABTS [μM TE/g]	murtilla non-ripe 878.18, murtilla ripe 405.76, blueberries Poland 265.92, murtilla-like berries non-ripe 244.22, chokeberry 152.63, blueberries Chile 150.45, raspberries 80.04, murtilla-like berries ripe 65.35	Arancibia-Avila et al. [89]
		FRAP [μM TE/g]	murtilla non-ripe 486.92, murtilla ripe 204.21, blueberries Poland 149.04, chokeberry 100.81, murtilla-like berries non-ripe 81.32, blueberries Chile 67.12, murtilla-like berries ripe 34.12, raspberries 33.98	
		CUPRAC [μM TE/g]	murtilla non-ripe 1012.42, murtilla ripe 507.89, blueberries Poland 265.76, chokeberry 215.85, murtilla-like berries non-ripe 203.83, blueberries Chile 141.36, murtilla-like berries ripe 92.36, raspberries 69.91	
Fruit and vegetable snacks extracts (chips and puffings)	Chips and puffings (100 g) were freeze dried, minced and macerated with 80% EtOH (500 mL) for 24 h at room temperature (repeated 3 times). Collected extracts were filtered, centrifuged, then the EtOH was evaporated. Powdered extracts were kept frozen until further use (−18 °C).	ABTS [mg TE/g DM extract]	chokeberry_puffing_ 37.44, blackcurrant_puffing_ 18.61, strawberry_puffing_ 16.16, apple-banana_chips_ 13.16, apple_chips_ 11.76, apple-blackcurrant_chips_ 10.74, apple-orange_chips_ 10.59, carrot_puffing_ 2.23	Gramza-Michałowska and Człapka-Matyasik [127]
		ABTS [EC_50_ mg/mL]	chokeberry_puffing_ 20.11, blackcurrant_puffing_ 38.92, strawberry_puffing_ 46.28, apple-banana_chips_ 55.62, apple_chips_ 64.79, apple-orange_chips_ 69.95, apple-blackcurrant_chips_ 71.92, carrot_puffing_ 437.12	
		DPPH [mg TE/g DM extract]	chokeberry_puffing_ 6.82, strawberry_puffing_ 6.40, apple-orange_chips_ 5.38, apple-blackcurrant_chips_ 5.31, apple-banana_chips_ 5.29, blackcurrant_puffing_ 5.28, apple_chips_ 5.02, carrot_puffing_ 3.47	
		DPPH [EC_50_ mg/ml]	chokeberry_puffing_ 10.04, strawberry_puffing_ 11.23, apple-orange_chips_ 12.67, blackcurrant_puffing_ 12.71, apple-banana_chips_ 13.11, apple-blackcurrant_chips_ 14.28, apple_chips_ 14.72, carrot_puffing_ 23.19	
Berry fruits	Homogenized fruits (50 g) were mixed with 150 mL 1% citric acid in H_2_O and extracted on an orbital shaker at 60ºC for 1 h.	ORAC [μmol TE/g DM extract]	elderberry 5783, blueberry 5646, chokeberry 5165, blackberry 4042, blackcurrant 3949	Denev et al. [36]
		TRAP [μmol TE/g DM extract]	chokeberry 4051, elderberry 3230, blueberry 2860, blackberry 2771, blackcurrant 2132	
		H-ORAC [μmol GAE/g DM extract]	blueberry 1293, chokeberry 1265, elderberry 1264, blackcurrant 874, blackberry 834	
		TBARS [inhibition of induced lipid peroxidation] [nmol/mL]	Chokeberry > elderberry > blackcurrant > blueberry > blackberry > control	
		NO scavenging activity time [sec]	blueberry 183, chokeberry 215, blackcurrant 280, elderberry 290, blackberry 363, control 947	
Fruit products: purees, concentrates, juices	Puree (1.5 g) was mixed with 5 mL H_2_O, and vortexed for 1 min. Afterwards, the solution was centrifuged at 3800 g for 5 min. The supernatants were collected in 20 mL volumetric flasks (procedure was repeated 3 times). After the last extraction, the flasks were filled up to the mark and aliquots of 1.5 mL were centrifuged.	FRAP [mmol Fe^2+^/100 g]	acerola 17.23, chokeberry 9.79, elderberry 9.33, boysenberry 5.90, blackcurrant 5.24, blackberry 4.53, açai-lime 4.09, lingonberry 3.90, strawberry 2.93, grape 2.91, cranberry 2.28, pomegranate 1.66, apple 1.33, orange 0.48	Müller et al. [128]
		ABTS [mmol TE/100 g]	acerola 10.57, chokeberry 9.73, elderberry 9.66, grape 5.92, blackcurrant 5.50, boysenberry 4.38, açai-lime 4.00, lingonberry 3.94, blackberry 3.08, cranberry 2.21, strawberry 2.08, pomegranate 1.75, apple 1.10, orange 0.40	
		ORAC [mmol TE/100 g]	chokeberry 11.45, elderberry 10.27, acerola 9.42, açai-lime 7.68, blackcurrant 6.99, boysenberry 5.31, strawberry 3.83, lingonberry 3.74, grape 3.49, blackberry 3.44, cranberry 2.57, pomegranate 2.50, apple 1.66, orange 1.07	
Chokeberry fruits- different cultivars	Fresh samples (10 g) were homogenized for 10 s in 100 mL of MeOH. The resulting paste was placed into Erlenmeyer flasks (120 mL) for 24 h at 25 °C, and the residue was then extracted with two additional portions of MeOH. The combined MeOH extracts were evaporated at 40 °C and redissolved in MeOH at a concentration of 100 mg/mL.	DPPH [g AAE/kg]	‘Viking’ 15.96, ‘Nero’ 15.32, ‘Hugin’ 11.15, ‘Aron’ 9.02, ‘Fertödi’ 8.89	Rop et al. [3]
		hydroxyl radical (OH^•^) scavenging activity [% inhibition]	‘Viking’ 34.15, ‘Nero’ 33.51, ‘Hugin’ 31.12, ‘Aron’ 25.01, ‘Fertödi’ 22.08	
		nitric oxide (NO^•^) scavenging activity [% inhibition]	‘Viking’ 41.46, ‘Nero’ 37.30, ‘Hugin’ 33.10, ‘Aron’ 28.42, ‘Fertödi’ 27.59	
		superoxide anion (O_2_^•−^) scavenging activity [% inhibition]	‘Viking’ 36.92, ‘Nero’ 35.96, ‘Hugin’ 30.48, ‘Aron’ 22.22, ‘Fertödi’ 21.24	
		lipid peroxidation (TBARS) [inhibition activity%]	‘Viking’ 19.81, ‘Nero’ 19.22, ‘Hugin’ 16.19, ‘Aron’ 12.57, ‘Fertödi’ 12.05	
Berry fruits	Ground berries (5 g) with 25 mL of MeOH at ambient temperature for 2 h with constant shaking. The solution was filtered, and the residue was repeatedly extracted with 20 mL of MeOH for 2 h. Finally, extracts were combined.	DPPH [inhibition%]	chokeberry var. ‘cleata’≥ chokeberry ‘Viking’≥ chokeberry ‘Aron’ > raspberry ‘Bristol’> raspberry ‘Meeker’> elderberry ‘Lacimiata’> raspberry ‘Poranna Rosa’> elderberry ‘Aurea’	Viskelis et al. [129]
Different berries	Berries (20 g) were grinded in MeOH (20 mL) acidified with HCl (0.1%). After 60 min the solution was filtered. The residue was extracted again, and the extracts were combined and diluted to volume of 50 mL with MeOH acidified with HCl (0.1%).	DPPH [inhibition %]	chokeberry > blackberry> red raspberry> strawberry	Jakobek et al. [74]
Fruit juices	Fruits (500 g) were thawed at room temperature, then processed in juice extractor natural fruit juice was centrifuged.	DPPH [μmol TE/mL]	chokeberry 72.44, elderberry 62.14, blackcurrant 30.15, sour cherry 12.52, blackberry 8.75, red raspberry 8.20, strawberry 4.39, sweet cherry 4.07	Jakobek et al. [74]
Fruit pomaces	The pomace was thawed and dried in the air dryer (50 °C, 2 h) before the analysis. Subsequently dried pomaces were ground and subjected to extraction in 80% EtOH.	ABTS [µM TE/g DM]	honeysuckle 62.24, blackcurrant 56.88, chokeberry 53.2, strawberry 23.32, Japanese quince 13.97	Nawirska et al. [119]
		DPPH [µM TE/g DM]	chokeberry 199.4, blackcurrant 138.81, honeysuckle 65.27, strawberry 58.67, Japanese quince 18.21	
		FRAP [µM Fe^3+^/g DM]	chokeberry 12.53, honeysuckle 11.13, Japanese quince 6.12, blackcurrant 5.24, strawberry 2.75	
Black chokeberry fruits, juice and pomace	Freeze-dried sample (1 g) was homogenized in 20 mL of MeOH. The slurry was filtered and filtrate was diluted in MeOH.	ABTS [μM TE/100 g DM]	pomace 779.58, fruits 439.49, juice 314.05	Oszmiański and Wojdyło [53]
		DPPH [μM TE/100 g DM]	pomace 301.89, fruits 279.38, juice 127.45	
Berry fruits	20 mL of MeOH/HCl 2% (95:5 *v*/*v*) were added to 20 g frozen berries. After 60 min, the berries were homogenized and centrifuged for 15 min at 3000 rpm. The supernatant solution was filtered under vacuum, and the residue was extracted again the same way. The solution was diluted to volume with MeOH/HCl 2%.	DPPH [EC_50_ mg fruit]	black currant ‘Tsema’ 1.0, black chokeberry ‘Nero’ 1.8, blackcurrant ‘Ben Lomond’ 1.8, blackcurrant ‘Silvergieters’ 2.5, blackcurrant ‘Burga’ 2.7, blackcurrant ‘Baldwin’ 2.9, blackcurrant ‘Tenah’ 3.4, blackcurrant ‘Noir De Bourgogne’ 3.6, blackcurrant ‘Black Down’ 4.2, redcurrant ‘Rotet’ 4.3, blackberry ‘Smoothstem’ 4.6, blackberry ‘Thornless Boy Sembes’ 5.2, raspberry ‘Sumner’ 5.5, blackberry ‘Black Diamond’ 5.7, blackberry ‘Darrow’ 5.7, red currant ‘Rosetta’ 5.7, red currant ’Red Lake’ 5.9, blackberry ‘Hull Thornless’ 6.2, blackberry ‘Chester’ 7.6, blackberry ‘Black Satin’ 9.5, raspberry ‘September’ 10.9	Benvenuti et al. [120]
Juice concentrates	Not specified.	ABTS [mg TE/mL]	blackcurrant 104.3, chokeberry 103.2, elderberry 98.7, redcurrant 36.0, strawberry 30.0, raspberry 24.7, red grape 23.1, cherry 18.7, plum 8.8	Bermúdez-Soto and Tomás-Barberán [87]
		DPPH [mg TE/mL]	chokeberry 60.0, blackcurrant 55.3, elderberry 43.3, redcurrant 23.1, strawberry 16.6, raspberry 13.4, red grape 10.4, cherry 10.0, plum 4.6	
Berry fruits	Sample (1 g) was mixed with 5 g of sea sand, transferred to a 22 mL extraction cell, and extracted with hexane/dichloromethane (1:1*v*/*v*) followed by acetone/water/acetic acid (70:29.5:0.5 *v*/*v*) extraction (ASE200). The extracts from hexane/dichloromethane were used to measure lipophilic ORAC_FL_, acetone/water/acetic acid extracts were used to measure the hydrophilic ORAC_FL_.	L-ORAC [μmol TE/g FW]	chokeberry 2.42, elderberry 1.97, red currant 1.27, blackcurrant ‘Titania’ 1.15, blackcurrant ‘Ben Alder’ 0.84, blackcurrant ‘Ben Nevis’ 0.75, blackcurrant ‘Ban Tirran’ 0.75, blackcurrant ‘Ben Lomond’ 0.68, blackcurrant ‘Ukarine’ 0.68, gooseberry ‘Marigold’ 0.45, gooseberry ‘Leveller’ 0.43, gooseberry ‘Careless’ 0.35, gooseberry ‘Dan’s Mistake’ 0.28, gooseberry ‘Whinham’ 0.15, gooseberry ‘Lancashine’ 0.15	Wu et al. [59]
		H-ORAC [μmol TE/g FW]	chokeberry 158.2, elderberry 145.0, blackcurrant ‘Ben Alder’ 100.6, blackcurrant ‘Ben Lomond’ 92.3, blackcurrant ‘Ben Nevis’ 90.6, blackcurrant ‘Ban Tirran’ 86.6, blackcurrant ‘Ukarine’ 53.7, blackcurrant ‘Titania’ 49.0, gooseberry ‘Lancashine’ 41.3, gooseberry ‘Whinham’ 39.2, gooseberry ‘Dan’s Mistake’ 37.1, gooseberry ‘Marigold’ 33.7, red currant 32.6, gooseberry ‘Leveller’ 26.4, gooseberry ‘Careless’ 20.4	
Berry fruits	Berries (3–5 g) were extracted twice with 10 mL of 80% acetone containing 0.2% formic acid using a Polytron for 2 min and then centrifuged. The supernatants were combined.	ORAC [μmol TE/g FW]	chokeberry 160.2, lingonberry ‘Amberland’ 38.1, blueberry ‘Serra’ 28.9, cranberry ‘Ben Lear’ 18.5	Zheng and Wang [130]

AAE: ascorbic acid equivalents, ACL: antioxidant capacity of lipid soluble compounds, ACW: antioxidant capacity of water soluble compounds, CUPRAC: cupric reducing antioxidant capacity, DM: dry matter, EC_50_: half maximum effective concentration, EtOH: ethanol, FRAP: ferric reducing antioxidant power, FW: fresh weight, GAE: gallic acid equivalents, H-ORAC: hydroxyl radical averting capacity, I_50_: extract concentrations which cause 50% inhibition, MeOH: methanol, ORAC: oxygen radical absorbance capacity, PCL: photochemiluminescence, TE: Trolox equivalents, TRAP: total peroxyl radical trapping parameter.
